# Dexmedetomidine Exerts Multi-level Effects to Ameliorate Alzheimer’s Disease Pathology in the Adult Zebrafish Brain

**DOI:** 10.1007/s12035-026-05906-9

**Published:** 2026-05-05

**Authors:** Dilek Nazli, Yusuf Kaan Poyraz, Kubilay Can, Dogac Ipekgil, Nilay Cakmak, Ebru Turhanlar-Sahin, Sevcan Hacoglu, Hale Aksu Erdost, Leyla Iyilikci, Gunes Ozhan

**Affiliations:** 1 Izmir Biomedicine and Genome Center, Dokuz Eylül University Health Campus, Inciralti-Balcova, 35340 Izmir, Turkey; 2 Department of Molecular Biology and Genetics, Izmir Institute of Technology, Urla , 35430 Izmir, Turkey; 3 Izmir International Biomedicine and Genome Institute, Dokuz Eylül University, Inciralti-Balcova, 35340 Izmir, Turkey; 4 Department of Anesthesiology and Reanimation, School of Medicine, Dokuz Eylül University, Izmir, 35340 Turkey; 5 Current Address: Louvain Institute of Biomolecular Science and Technology, Université Catholique de Louvain, Louvain-La-Neuve, Belgium

**Keywords:** Alzheimer’s disease, Dexmedetomidine, β-Amyloid, Neuroinflammation, Astrogliosis, Neurogenesis, Neuroprotection, Zebrafish

## Abstract

Alzheimer’s disease (AD) is a complex neurodegenerative condition involving β-amyloid (Aβ) deposition, tau abnormalities, neuroinflammation, neuronal degeneration, and progressive impairment of cognitive functions. Despite extensive research, effective disease-modifying therapies remain limited, highlighting the need for translationally relevant models and repurposable therapeutic candidates. Dexmedetomidine (DEX), an α2-adrenergic receptor agonist with known neuroprotective properties, was investigated in an adult zebrafish model of AD established through cerebroventricular administration of Aβ42. DEX treatment significantly reduced Aβ accumulation and was associated with reduced amyloidogenic gene expression, indicating transcriptional changes in amyloidogenic pathway-related genes. DEX attenuated neuroinflammation by reducing glial activation, lowering pro-inflammatory cytokine gene expression, and increasing expression of the anti-inflammatory gene *il10*. Immunofluorescence assessment further demonstrated reduced astrogliosis and preserved neuronal marker integrity, as indicated by increased HuC/D levels. Interestingly, DEX attenuated Aβ-induced proliferative responses, characterized by decreased PCNA expression, while enhancing cleaved caspase-3 levels, suggesting changes in proliferation and apoptotic signaling under Aβ stress conditions. Behavioral assessments further demonstrated that DEX alleviated Aβ42-induced anxiety- and aggression-like behaviors, improving behavioral phenotypes in this model. Overall, these findings underscore the multi-level effects of DEX in modulating AD-related pathological features. As a clinically available agent, DEX represents a promising candidate for repurposing in neurodegenerative disease contexts. Further preclinical studies in mammalian models are warranted to validate its translational relevance and therapeutic potential.

**Supplementary Information:**

The online version contains supplementary material available at 10.1007/s12035-026-05906-9.

## Introduction

AD is the most prevalent neurodegenerative disorder that affects millions of individuals worldwide with an increasing prevalence [1–3]. The key neuropathological signatures of AD involve extracellular deposits of Aβ plaques, intraneuronal neurofibrillary tangles formed by hyperphosphorylated tau, extensive synaptic impairment, and progressive neuronal degeneration, most prominently affecting the hippocampus and cortex. On the molecular level, abnormal cleavage of amyloid precursor protein (APP) by β-secretase (BACE1) and γ-secretase generates Aβ peptides with high aggregation potential, which accumulate within brain tissue in a toxic manner [4–7]. This accumulation triggers a cascade of cellular disturbances, including oxidative stress, mitochondrial dysfunction, disrupted calcium homeostasis, and dysregulation of multiple signaling pathways [8–10], [11]. Concurrently, tau hyperphosphorylation destabilizes microtubules and impairs axonal transport, further contributing to neuronal degeneration [12–14].

Beyond Aβ and tau abnormalities, persistent neuroinflammation has been recognized as a major contributor to AD progression. This process is characterized by the activation of glial populations, particularly microglia and astrocytes, which secrete pro-inflammatory mediators and reactive oxygen species, thereby intensifying neuronal damage and sustaining a chronic cycle of inflammation [15, 16], [17, 18]. The inflammatory microenvironment disrupts synaptic function, worsens neuronal injury, and accelerates neurodegeneration. These converging molecular and cellular alterations indicate that AD is not solely a proteinopathy but also a complex, glia-mediated inflammatory disorder.

The multifactorial nature of AD pathogenesis has limited the success of single-target therapies. Current treatments provide only symptomatic relief and do not halt disease progression, highlighting the need for multi-level therapeutic strategies [19–22]. In this context, compounds with anti-inflammatory and neuroprotective properties are gaining interest [23–25]. Dexmedetomidine (DEX), a clinically established α2-adrenergic receptor agonist used for sedation and analgesia, can cross the blood–brain barrier, and has shown neuroprotective effects in various models [26–31]. In the present study, this property is particularly relevant, as retro-orbital injection was employed as a systemic delivery route to enable central nervous system (CNS) exposure [32]. Through its central actions, DEX reduces neuronal apoptosis, mitigates excitotoxicity, and alleviates neuroinflammatory responses, positioning it as a promising candidate for neurodegenerative diseases such as AD  [33–36].

Zebrafish (*Danio rerio*) have become a valuable model for AD research owing to their genetic homology with humans and the conservation of major AD-related genes such as *APP*, *PSEN1*, *PSEN2*, *MAPT*, and *APOE* and their neuroanatomical and neurochemical characteristics comparable to the human brain [6, 37–41]. This homology enables the study of disease-relevant mechanisms in a vertebrate system. Both transgenic models that enable stable expression of human AD-related genes and models induced via cerebroventricular microinjection of Aβ peptides have been developed, offering complementary approaches to mimic AD pathology and progression [42–44]. Together with well-established behavioral assays, these models offer a versatile and scalable in vivo platform for evaluating neurodegeneration and therapeutic interventions. Importantly, zebrafish enable the investigation of Aβ-induced neuroinflammatory responses and associated behavioral alterations in a whole-organism context [32, 45]. However, it should be noted that zebrafish models do not fully recapitulate the chronic and progressive nature of human AD pathology.

Here, we assessed the potential of DEX to counteract Aβ42-driven neurotoxicity using an adult zebrafish model of AD. Our results suggest that DEX improves outcomes at the molecular, cellular, and behavioral levels. Specifically, DEX was found to limit Aβ deposition, induce transcriptional changes in amyloidogenic pathway genes, and be associated with changes in neuroinflammatory responses, including reduced pro-inflammatory cytokine expression and increased anti-inflammatory signaling. DEX also diminished astrogliosis and is associated with reduced glia-related neurotoxicity, with preservation of neuronal marker expression as indicated by increased HuC/D expression. Interestingly, while DEX reduced degeneration-associated proliferation, it enhanced apoptotic signaling, suggesting changes in cellular turnover dynamics. These molecular and cellular effects translated into significant behavioral improvements. Overall, our findings highlight the anti-inflammatory and neurobehavioral effects of DEX and support its potential as a promising therapeutic agent for AD.

## Materials and Methods

### Ethics

Animal experiments were performed in accordance with the European Union Directive 2010/63/EU on the care and use of laboratory animals. The study protocol received ethical approval (Approval ID: 2020-007) from the Local Animal Ethics Committee of the Izmir Biomedicine and Genome Center (IBG-AELEC), İzmir, Türkiye, on 12 February 2020.

### Zebrafish Husbandry

Wild-type AB strain zebrafish (6–10 months old) were obtained from the Zebrafish Core Facility at the Izmir Biomedicine and Genome Center (IBG). Fish were maintained at 28.5 °C under a 14 h light/10 h dark cycle in accordance with IBG Animal Care and Use Committee guidelines.

### Toxicity Assay

Dexmedetomidine HCl (AdooQ BioScience, A11846; CA, USA) was initially dissolved in sterile distilled water to generate a stock solution and subsequently diluted to the required concentrations immediately before administration. A toxicity assessment was performed to identify the maximum non-toxic dose of DEX appropriate for application in zebrafish. The assay was performed using dechorionated larvae at 48 h post-fertilization (hpf), individually housed in 96-well plates at 28.5 °C [46]. Larvae were treated with two different concentrations of DEX in E3 embryo medium (5 mM NaCl, 0.17 mM KCl, 0.33 mM CaCl₂, 0.33 mM MgSO₄ in dH₂O). Larvae were maintained at 28.5 °C, and the E3 medium containing DEX was refreshed daily to ensure stable exposure. Larval development and survival were evaluated at 24- and 48-h post-treatment using a stereomicroscope (SZX2 Series, Olympus, Tokyo, Japan), and digital images were acquired during analysis [47, 48]. Each treatment group consisted of *n* = 30 larvae. The experiment was concluded after 48 h of DEX exposure.

### Peptide Preparation

The Aβ42 peptide (DAEFRHDSGYEVHHQKLVFFAEDVGSNKGAIIGLMVGGVVIA) synthesized by PeptiTeam (Ankara, Türkiye), was initially dissolved at 20 mg/mL in a 1:1 solution of acetonitrile and deionized water [42, 43].

### Sample Size Determination

To ensure that each experimental group had an adequate number of biological replicates, a priori power analysis was performed using G*Power software version 3.1.9.7. The analysis was conducted with an effect size of 0.8, an alpha level (*α*) of 0.05, and a statistical power (1 − β) of 0.8, as previously recommended [49]. Based on these parameters, the required sample size was calculated to be six for each group. The effect size was estimated from preliminary pilot experiments conducted under the same experimental conditions, based on consistent observations across multiple experimental endpoints. Molecular (qRT-PCR) and imaging (immunofluorescence) analyses were performed on separate cohorts of animals to avoid cross-interference between assays.

### Cerebroventricular Microinjection of Aβ

Adult zebrafish (6–10 months old, 25–32 mm in length) were anesthetized using system water containing tricaine (MS-222, 50 mg/mL) in accordance with previously established protocols [32, 42, 43] [50–52]. The chosen Aβ42 concentration (40 μM) was guided by prior studies in adult zebrafish and was sufficient to induce robust neurotoxic effects while preserving overall viability. Post-injection, fish were returned to fresh system water and monitored for recovery. Injection placement was confirmed by co-injection of a fluorescent tracer (CM-DiI; Thermo Fisher Scientific, MA, USA). The water was renewed daily. A 3-day incubation period was allowed for the establishment of Aβ-induced toxicity. Each experimental group comprised 6 fish (*n* = 6).

### Retro-Orbital Injection of DEX

To assess the therapeutic effects of DEX, adult zebrafish were randomly assigned to three groups (*n* = 6 per group; 3 males and 3 females). Retro-orbital (RO) injection was selected as an effective method for systemic delivery and penetration across the blood–brain barrier [53]. Injections were performed under anesthesia (tricaine, MS-222), and injection placement was confirmed by co-injection of a fluorescent tracer (DiO; Thermo Fisher Scientific, MA, USA). Fish were monitored during recovery until normal swimming behavior was observed, and no procedure-related mortality or adverse effects were detected. The “Control” group received PBS via both CVMI and RO injection and underwent all procedural steps, including anesthesia and sham handling, to control for procedure-related stress. The “Control + DEX” group received PBS via CVMI and 2 µL of 250 μM DEX via RO injection. The “Aβ-induced toxicity model” group received Aβ42 via CVMI, followed by PBS via RO injection. The “Aβ-induced toxicity model + DEX” group received Aβ42 via CVMI and 2 µL of 250 µM DEX via RO injection.

### Tissue Preparation and Cryosectioning

Zebrafish were sacrificed by rapid cooling in ice-cold water, after which the heads of three animals from each group (*n* = 3) were separated and immersed in 4% paraformaldehyde (PFA) prepared in PBS at 4 °C for 48 h [54, 55]. The preserved samples were subsequently decalcified for 2 days in a mixture of 20% sucrose and 20% EDTA at 4 °C, followed by an additional 2-day incubation in 30% sucrose with 20% EDTA. Afterwards, tissues were embedded in a medium consisting of 20% sucrose and 7.5% gelatin, rapidly frozen on dry ice, and stored at –80 °C. Serial transverse sections of 14 µm were cut on a cryostat (Leica CM1850, Wetzlar, Germany), kept at 65 °C for 48 h, and then transferred to –20 °C until further use.

### Immunofluorescence Staining and Imaging

Cryosections stored at –20 °C were brought to room temperature and left to air-dry for 20 min. Tissue permeabilization was achieved by washing the sections twice in PBSTX (PBS with 0.3% Triton X-100) for 10 min each, followed by two additional washes in PBS for 5 min [54, 56]. Antigen retrieval was conducted by immersing the samples in 10 mM sodium citrate at 85 °C for 15 min. After cooling, the slides were rinsed again in PBS (2 × 5 min) and PBSTX (2 × 10 min). Subsequently, sections were incubated overnight at 4 °C with primary antibodies diluted in PBSTX: rabbit anti-β-Amyloid (1:500; Cat# D54D2; Cell Signaling Technology, MA, USA), mouse anti-HuC/HuD (1:500; Cat# A-21271; Thermo Fisher Scientific, MA, USA), mouse anti-GFAP (1:500; Cat# ab154474; Abcam, Cambridge, UK), rabbit anti-L-Plastin (1:200; Cat# 55208−1-AP; Proteintech, CA, USA), rabbit anti-cleaved caspase-3 (1:300; Cat# 9664S; Cell Signaling Technology, MA, USA), and mouse anti-PCNA (1:500; Cat# M087901-2, Agilent Technologies, CA, USA).

After incubation with the primary antibodies, sections were rinsed three times in PBSTX for 10 min each. They were then exposed to secondary antibodies for 2 h at room temperature. The secondary antibodies applied were donkey anti-rabbit IgG, Rhodamine (TRITC)-conjugated (1:500; Cat# 711–025–152; Jackson ImmunoResearch Laboratories, PA, USA) and donkey anti-mouse IgG (H + L), Cy5-conjugated (1:500; Cat# 712–175-150; Jackson ImmunoResearch Laboratories, PA, USA). Nuclear staining was performed with 4′,6-diamidino-2-phenylindole (1 µg/ml; DAPI; Cat# 4083S; Cell Signaling Technology, MA, USA). All primary antibodies used in this study were validated based on manufacturer-provided information and further confirmed in zebrafish tissue under our experimental conditions (Table S2). After final PBS washes, slides were mounted using 70% glycerol.

Confocal images were acquired on an LSM 980 microscope (Zeiss) at 22 °C using a 63 × oil immersion objective (NA 1.4), with laser power set to 2.5%, a pinhole size of 1 AU, and a pixel size of 0.198 × 0.198 µm [32, 57]. Excitation wavelengths were 340 nm for DAPI (emission 430–480 nm), 561 nm for the green channel (emission 570–620 nm), and 650 nm for the red channel (emission 660–750 nm). Images were captured and analyzed using Zen Blue software (Zeiss AG, Oberkochen, Germany). Image quantification was performed in a blinded manner, with investigators unaware of the experimental group assignments. Regions of interest (ROIs) were defined based on anatomical landmarks within the telencephalon and applied consistently across all samples. Fluorescence intensity and positive cell counts were quantified manually using ImageJ (NIH, USA), with identical threshold settings applied across all groups. Fluorescence intensity was measured as mean signal per unit area within each ROI, following background subtraction, and was therefore normalized to area.

### RNA Isolation and cDNA Synthesis

At 48 h post-RO injection, zebrafish were euthanized in ice-cold water. The telencephalon was dissected from pooled brain tissue (*n* = 3 per group, each biological replicate consisting of pooled tissue from three individuals). Total RNA was isolated with the miRNeasy Mini Kit (Qiagen, Hilden, Germany) following the protocol provided by the manufacturer. Complementary DNA (cDNA) was subsequently generated using the iScript Reverse Transcription Supermix for RT-qPCR (Bio-Rad Laboratories, Inc., CA, USA) [58].

### Quantitative Real-Time PCR and Statistical Analysis

qRT-PCR analysis was conducted in three technical replicates using the GoTaq qPCR Master Mix (Promega, WI, USA) on an Applied Biosystems 7500 Fast Real-Time PCR instrument (Thermo Fisher Scientific, MA, USA), following the procedure outlined previously  [59]. To quantify expression, values were normalized to the housekeeping gene *rpl13a*, and fold changes were determined through the ΔΔCt approach. Primer amplification efficiencies were routinely assessed and confirmed to be within an acceptable range for the ΔΔCt method. Data derived from three independent biological samples were processed in GraphPad Prism 8 (GraphPad Software Inc., CA, USA) and are presented as mean ± SD [60]. Group differences were evaluated using one-way analysis of variance (ANOVA). Primer sequences for *appa*, *appb*, *bace*, *psen1*, *psen2*, *il1β*, *tnfα*, *il6st*, *il10*, and *rpl13a* are summarized in Table S1.

### Behavioral Tests and Statistical Analysis

Behavioral assessments were conducted 48 h post-RO injection, immediately prior to tissue collection. Prior to testing, fish were acclimated to the experimental room conditions for at least 30 min. Each zebrafish was placed individually into a trapezoidal test tank (27 × 10 × 17 cm) containing 3.5 L of system water. Behavior was recorded using two synchronized video cameras. For the mirror-biting assay, mirror position, tank background, and lighting conditions were kept constant across all trials to ensure standardized testing conditions. To reduce the impact of circadian rhythms, all experiments were carried out between 9:00 a.m. and 2:00 p.m. Between trials, the tank water was refreshed to eliminate stress-related artifacts [61]. Water temperature was maintained at 27 ± 1 °C throughout the testing period. Behavioral recordings were analyzed using Panlab SMART v3.0 software (Harvard Apparatus, MA, USA). Each experimental group comprised 10 fish (*n* = 10) including an equal number of males and females (5 males and 5 females per group). The same fish were used across all behavioral assays. Sex was not included as a factor in statistical analyses. Both sexes were included to account for potential sex-dependent variability. All statistical analyses were performed using R version 4.5.1 [62]. Normality and homogeneity of variances were assessed using the Shapiro–Wilk and Levene’s tests, respectively. For datasets not involving repeated measurements, group differences were evaluated using one-way ANOVA; when the assumption of homogeneity of variances was violated, Welch’s ANOVA was applied. Post hoc comparisons were conducted using Tukey’s HSD test for standard ANOVA and Games–Howell test for Welch’s ANOVA. For repeated-measures behavioral data obtained from the novel tank diving test, linear mixed-effects models (LMMs) were fitted using the lme4 package [63], with Group, Time, and their interaction (Group × Time) included as fixed effects, and individual fish ID included as a random intercept. Type III analysis of variance [64] tables with Satterthwaite-adjusted degrees of freedom were generated using the lmerTest package [64]. Planned pairwise comparisons were conducted using estimated marginal means and the emmeans package with Sidak correction for multiple testing  [65]. Multiple comparison corrections were applied within each behavioral test; however, different behavioral endpoints were analyzed as independent outcomes and were not subjected to global correction across endpoints. All experimental subjects were randomly assigned to treatment groups to minimize allocation bias, and blinding was maintained during both experimental procedures and statistical analyses. For each comparison, the test statistics, degrees of freedom, and exact *p*-values were calculated and reported. Complete details of all statistical tests are provided in Table S3. Trajectory plots derived from behavioral tracking analyses are provided in Table S4.

### Novel Tank Diving Test

The novel tank diving test is a well-established behavioral assay for assessing anxiety-like behavior and locomotor activity in zebrafish based on their responses to an unfamiliar environment [66, 67]. Each test tank was filled with 15 cm of system water and vertically divided into three equal horizontal zones: bottom, middle, and top [54]. The test was initiated immediately after each fish was gently introduced into the tank and continued uninterrupted for 30 min. Behavioral data were analyzed using Panlab SMART v3.0 software, with the following parameters evaluated: distance traveled in the bottom and top zones, latency to top zone, and mean speed in the bottom zone. Each experimental group comprised 10 fish (*n* = 10).

### Mirror Biting Test

The mirror biting test is a widely used assay to evaluate aggression-like behavior in zebrafish. Due to the lack of self-recognition, zebrafish typically perceive their mirror image as a conspecific intruder, triggering social challenge responses. This perception induces a range of aggressive behaviors, including mirror approach, fin spreading, rapid swimming, and sudden changes in orientation toward the reflection [68, 69]. These behaviors provide valuable insights into aggression-related motivation and the neurobiological mechanisms underlying social recognition, making the mirror test a valuable tool for evaluating behavioral phenotypes linked to neuropsychiatric and neurodegenerative conditions. Before testing, the mirror was covered with an opaque sheet, and a 5-min habituation period commenced once the fish was introduced into the tank. After habituation, the cover was removed, and video recording was initiated simultaneously. Each fish was observed for 15 min without disturbance. Behavioral parameters analyzed included distance traveled (cm), time spent (s), and average speed (cm/s) within the mirror-biting zone, as well as average speed (cm/s) across the entire tank. Each experimental group comprised 10 fish (*n* = 10).

## Results

### Effects of DEX Exposure on Survival and Morphology in Zebrafish Larvae

**Fig. 1:**
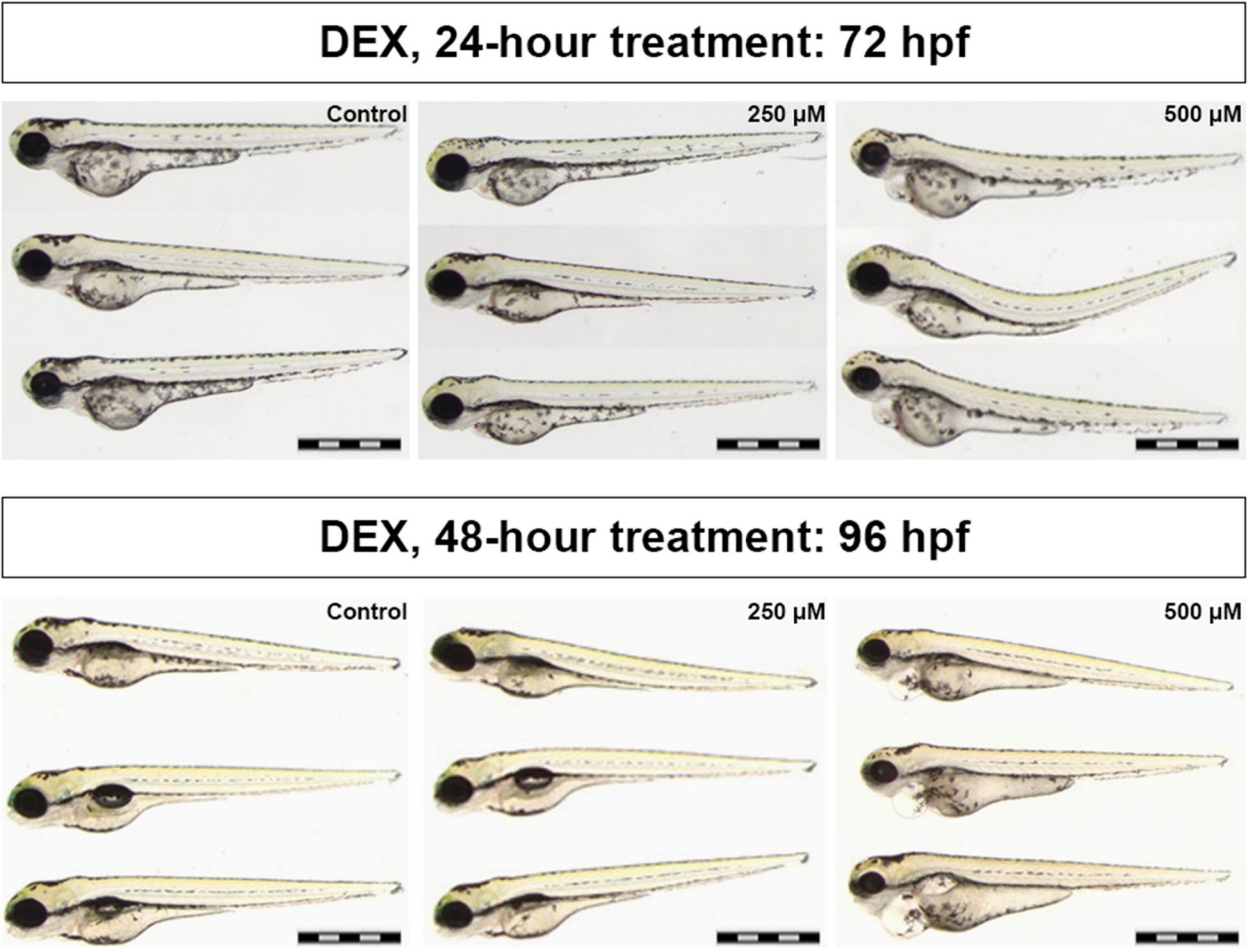
Effects of DEX exposure on survival and morphology in zebrafish larvae. Representative bright field images of zebrafish larvae treated with dexmedetomidine (DEX) at 250 µM and 500 µM concentrations. Treatments were initiated at 48 h post-fertilization [73] and lasted for either 24 h (top panel, imaged at 72 hpf) or 48 h (bottom panel, imaged at 96 hpf). Untreated larvae served as controls. While larvae treated with 250 µM DEX exhibit normal morphology, those exposed to 500 µM show clear developmental abnormalities, including pericardial cardiac edema and spinal curvature. Based on these observations, 250 µM was selected as the working dose for subsequent experiments. Scale bar, 1 mm. Images are representative of at least three independent experiments. *n* = 30 larvae per group

While low to moderate doses of DEX have demonstrated protective effects in rat models of endotoxin-induced sepsis, high-dose administration has been associated with significant toxicity, with a reported lethal dose 50% (LD₅₀) within the first 24 h in mice [70], [71, 72]. To evaluate DEX toxicity and determine a safe working concentration in zebrafish, we evaluated two concentrations (250 µM and 500 µM) in 48 hpf larvae. The larvae were monitored at 24- and 48-h post-treatment (hpt) for survival and morphological changes. Treatment with 250 µM DEX resulted in 100% survival, with no observable developmental defects throughout the 48-h observation period (Fig. 1). In contrast, exposure to 500 µM DEX induced notable developmental defects by 24 hpt, including spinal curvature and mild cardiac and yolk sac edema, which progressed to severe cardiac edema and a 40% mortality rate by 48 hpt. Based on these findings, 250 µM DEX was identified as a non-toxic concentration under the conditions tested and was therefore selected for use in subsequent experiments. This concentration was selected based on larval toxicity assessment to define a safe exposure range and was subsequently applied in adult experiments. It should be noted that toxicity assessments in larvae may not directly reflect therapeutic exposure or pharmacological responses in adult zebrafish.

### DEX Is Associated with Changes in Aβ Accumulation and Amyloidogenic Gene Expression

**Fig. 2:**
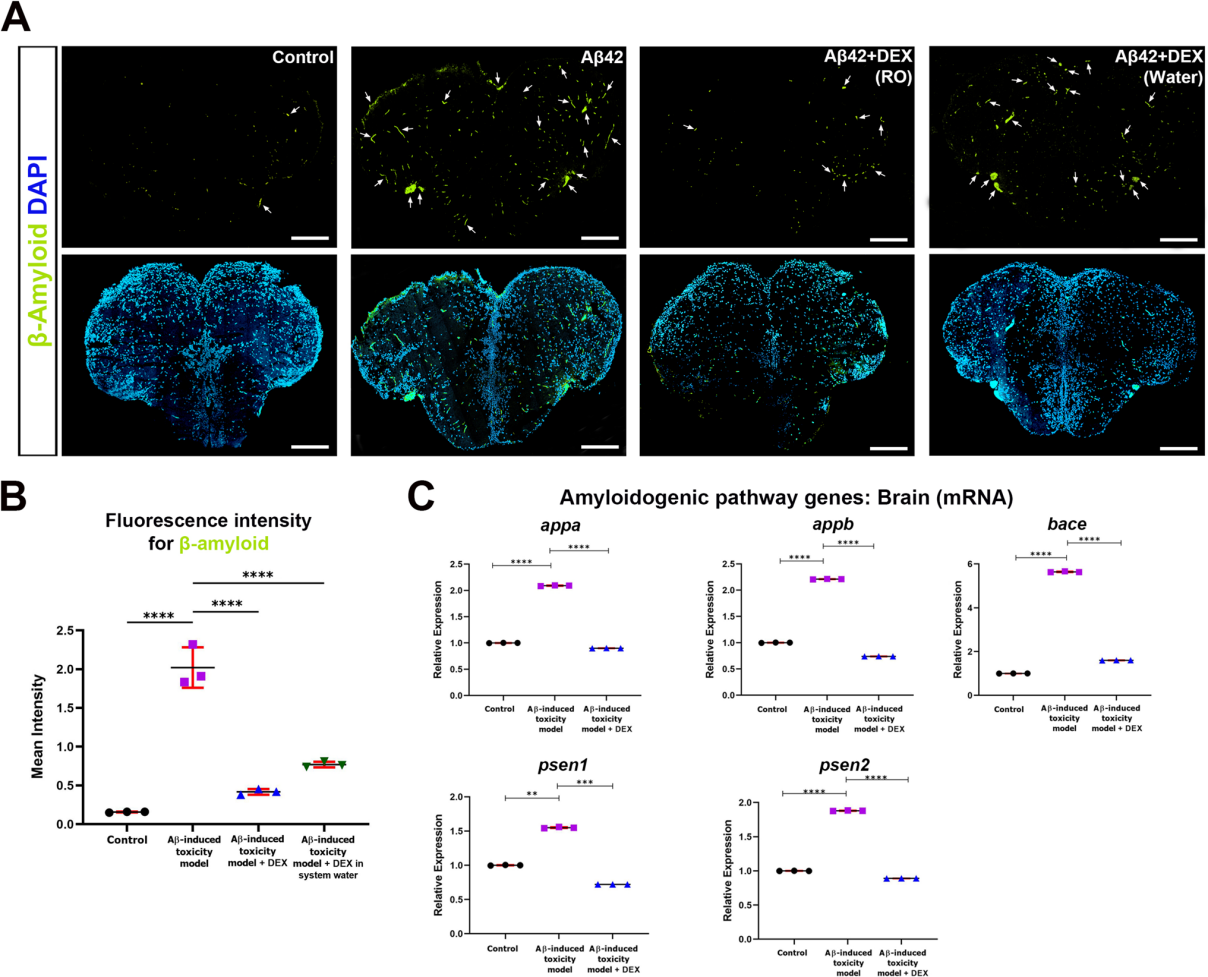
Aβ accumulation and amyloidogenic gene expression in response to DEX treatment. **A** Representative immunofluorescence images of zebrafish telencephalon sections showing β-amyloid (Aβ) deposition (green), using an anti-Aβ antibody, with nuclear counterstaining using 4′,6-diamidino-2-phenylindole (DAPI, blue). Sections are from the four groups: control, Aβ42-injected group, Aβ42-injected co-treated with dexmedetomidine (DEX) via retro-orbital (RO) injection, and Aβ42-injected co-treated with DEX administered through system water. Aβ42 injection induces marked Aβ plaque accumulation (white arrows), which is significantly reduced upon DEX (RO) treatment. DEX administration via system water does not result in a significant reduction in Aβ levels. Scale bar, 200 µm. Images are representative of at least three independent experiments. **B** Quantification of Aβ fluorescence intensity in telencephalon sections. Aβ42 injection significantly increases Aβ accumulation compared to control, while DEX (RO) co-treatment significantly reduces Aβ levels. No statistically significant reduction is observed in the DEX (Water) group. Data are presented as mean ± SD (*n* = 4 biological replicates per group). Statistical significance was determined using one-way analysis of variance (ANOVA). ****p < 0.0001. **C** Relative mRNA expression levels of key amyloidogenic pathway genes (*appa*, *appb*, *bace*, *psen1*, and *psen2*) in zebrafish brains. Aβ42 injection significantly upregulates all genes assessed, while DEX (RO) co-treatment markedly downregulates their expression. Data are presented as mean ± standard deviation (SD) (*n* = 3 biological replicates, each measured in triplicate). Statistical significance was determined using one-way ANOVA. **p < 0.01, ***p < 0.001, ****p < 0.0001

CVMI of Aβ42 successfully induced Aβ pathology in the adult zebrafish brain, as previously demonstrated [32, 50]. In zebrafish, pharmacological agents are typically administered via immersion, microinjection, or oral routes [74]. To validate the Aβ-induced toxicity model and compare the efficacy of two DEX delivery methods, Aβ42-injected zebrafish were treated either by RO injection or by adding DEX directly to the system water. Immunofluorescence staining for Aβ revealed no Aβ deposition in the control group (Fig. 2A). In contrast, extensive Aβ accumulation was observed in the brains of Aβ42-injected, untreated fish. Notably, DEX treatment via RO injection markedly reduced Aβ-associated immunofluorescence signal compared to the untreated Aβ group, indicating a significant reduction in Aβ levels under the conditions tested. While DEX administered via immersion also had a measurable impact on Aβ levels, it resulted in a less pronounced improvement compared to RO-administered DEX. Quantitative analysis of Aβ fluorescence intensity confirmed a significant increase in signal following Aβ42 injection, which was significantly reduced by both RO- and immersion-administered DEX, with a stronger effect observed in the RO group (Fig. 2B). DEX treatment in the Control + DEX group did not result in statistically significant changes in Aβ levels compared to the controls, indicating no effect on basal Aβ levels under non-pathological conditions (Figure S1A). Since Aβ accumulation is known to amplify amyloidogenic processing through a feed-forward mechanism involving APP and BACE1 [75], we next analyzed the expression of genes associated with this pathway. Aβ42 exposure significantly upregulated the zebrafish homologs of APP—*appa* and *appb*—as well as key components of the β- and γ-secretase complexes, including *bace*, *psen1*, and *psen2* (Fig. 2C). RO administration of DEX was associated with a reduction in this transcriptional response. Expression levels of *appa*, *appb*, *psen1*, and *psen2* were significantly downregulated, even falling below baseline levels observed in controls. Notably, *bace* expression returned to near-control levels, suggesting that DEX may be associated with modulation of pathway-related gene expression and may influence amyloidogenic processes at the transcriptional level. Importantly, no significant changes in Aβ-related gene expression were observed in the Control + DEX group, indicating no effect under basal conditions (Figure S2). Together, these findings demonstrate that DEX, when delivered via RO injection, reduces Aβ accumulation and is accompanied by transcriptional changes in amyloidogenic pathway-related genes. These observations support a potential role for DEX in modulating Aβ-related pathology in vivo.

### DEX Contributes to the Modulation of Inflammatory Responses

**Fig. 3:**
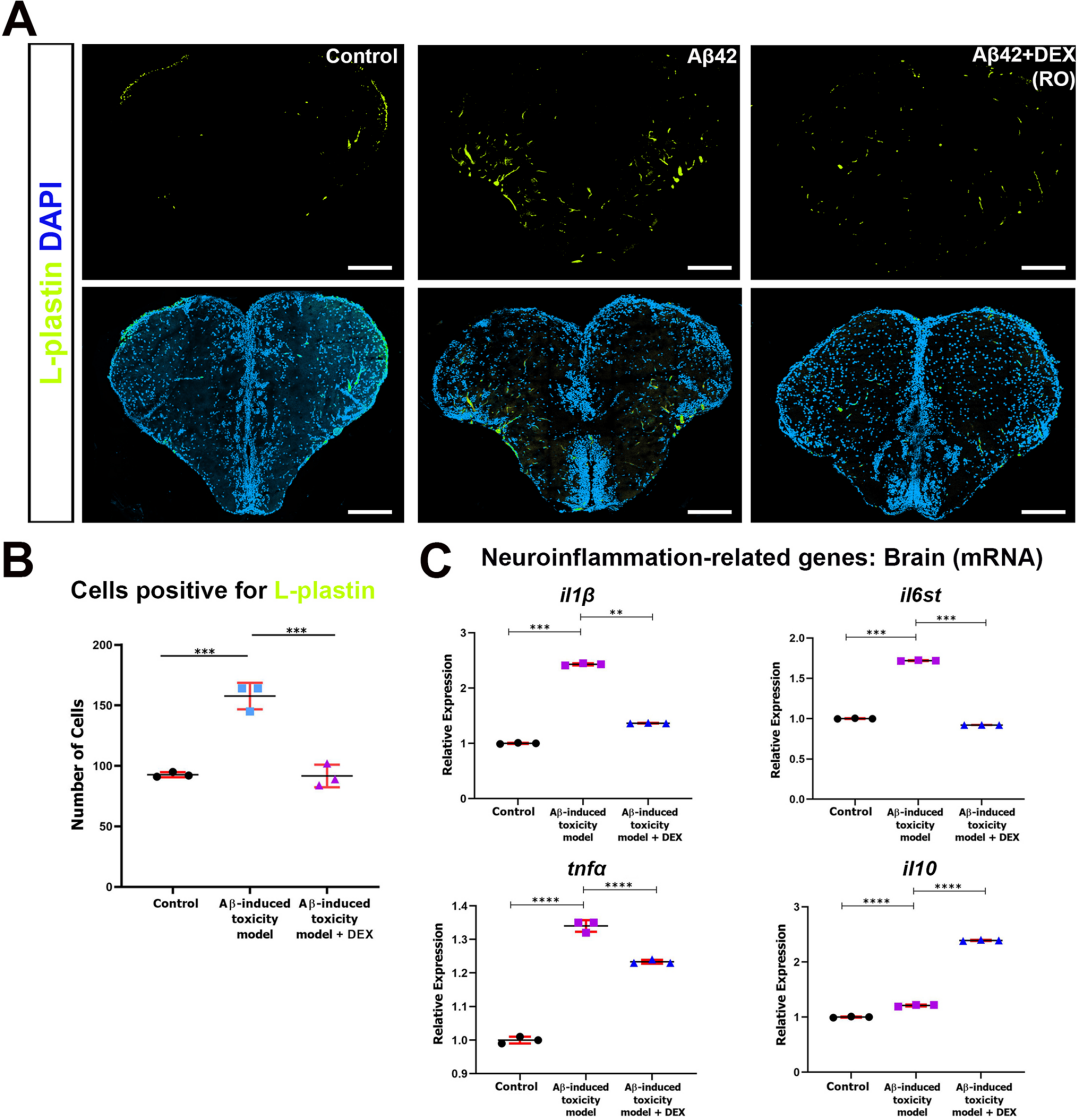
Inflammatory gene expression and immune cell responses following DEX treatment. **A** Representative immunofluorescence images of zebrafish telencephalon sections stained for L-plastin (green), a marker of activated microglia, and 4′,6-diamidino-2-phenylindole (DAPI, blue) for nuclear counterstaining. Baseline L-plastin expression is minimal in control animals but markedly elevated following β-amyloid42 (Aβ42) administration, indicating glial activation. Dexmedetomidine (DEX) treatment markedly reduces L-plastin signal, suggesting a suppression of the neuroinflammatory response. Scale bar, 200 µm. Images are representative of at least three independent experiments. **B** Quantification of L-plastin-positive cells. Aβ42 significantly increases the number of L-plastin-positive cells, whereas DEX treatment reduces this number to near-control levels. Data are presented as mean ± standard deviation (SD) (*n* = 4 biological replicates per group). Statistical significance was determined using one-way analysis of variance (ANOVA). ***p < 0.001. **C** Relative mRNA expression levels of pro-inflammatory (*il1b, il6st, tnfα*) and anti-inflammatory (*il10*) cytokines in whole-brain samples. Aβ42 significantly upregulates pro-inflammatory gene expression; the increase in *il10* is not significant. DEX treatment significantly downregulates *il1b, il6st*, and *tnfa*, while significantly upregulating il10. Data are presented as mean ± SD (*n* = 3 biological replicates, each measured in triplicate). Statistical significance was determined using one-way ANOVA. **p < 0.01, ***p < 0.001, ****p < 0.0001

L-plastin (LCP1) is an actin-bundling protein expressed in hematopoietic cells where it plays a pivotal role in immune cell activation, migration, and phagocytosis. Increasing evidence also supports its expression in the CNS, particularly in activated microglia [76]. Suppression of L-plastin has been proposed as a therapeutic approach to attenuate neuroinflammation in neurodegenerative diseases such as Parkinson’s disease [77]. To evaluate the neuroinflammatory response elicited by Aβ42 and the anti-inflammatory potential of DEX, we performed immunofluorescence staining with for L-plastin in brain sections from control, Aβ42-injected, and DEX-treated zebrafish. Confocal microscopy revealed a marked increase in L-plastin expression in the Aβ42-injected group compared to the control group, indicating increased immune cell activation, including microglia (Fig. 3A). Notably, DEX treatment substantially reduced L-plastin expression. Quantification of L-plastin-positive cells confirmed a significant increase in the Aβ42 group, which was effectively reversed by DEX treatment, returning to control levels (Fig. 3B). In contrast, L-plastin expression in the Control + DEX group remained comparable to control levels, suggesting that DEX does not induce microglial activation in the absence of pathology (Figure S1B). To further characterize the inflammatory profile, we analyzed the expression of key pro-inflammatory cytokines (*il6st*, *tnfa*, and *il1b*) and the anti-inflammatory cytokine *il10* using qRT-PCR. Aβ42 injection induced robust upregulation of all three pro-inflammatory genes, consistent with activation of a neuroinflammatory response (Fig. 3C). DEX treatment significantly suppressed *il6st* and *il1b* expression, indicating reduced pro-inflammatory gene expression. Importantly, while *il10* levels remained unchanged following Aβ42 injection, DEX treatment significantly enhanced *il10* expression, indicating a change in inflammatory gene expression, including upregulation of an anti-inflammatory cytokine. No statistically significant changes in the expression of inflammation-related genes were observed in the Control + DEX group compared to the Control group, indicating that DEX does not alter basal inflammatory signaling under non-pathological conditions (Figure S2). Thus, these results demonstrate that DEX alleviates Aβ42-induced neuroinflammation by reducing L-plastin-positive immune cell activation and altering inflammatory gene expression profiles. This gene expression–based evidence underscores the therapeutic potential of DEX in targeting inflammatory pathways implicated in AD.

### DEX Drives Changes in Apoptotic and Proliferative Responses to Aβ Toxicity

**Fig. 4:**
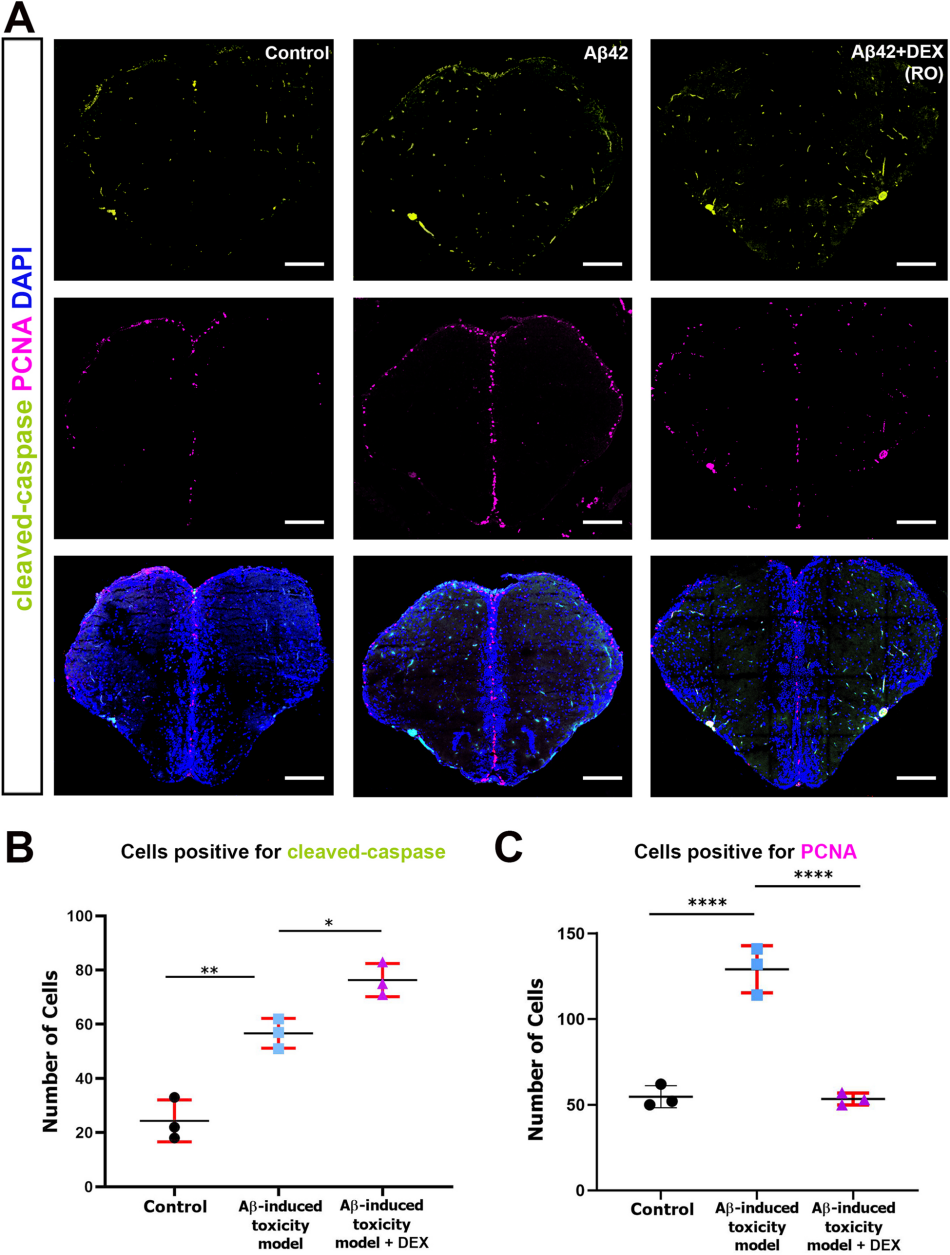
Apoptotic and proliferative responses in Aβ-treated zebrafish following DEX treatment. **A** Representative immunofluorescence images of zebrafish telencephalon sections stained for cleaved caspase-3 (green; apoptosis marker), proliferating cell nuclear antigen (PCNA; magenta; proliferation marker), and 4′,6-diamidino-2-phenylindole (DAPI; blue; nuclei). β-Amyloid42 (Aβ42) exposure increases levels of cleaved caspase-3, a marker of apoptosis, and this response is further enhanced by DEX treatment, indicating increased apoptotic signaling. In contrast, Aβ42 significantly elevates PCNA expression, indicative of aberrant proliferative activity, which is markedly reduced following dexmedetomidine (DEX) administration. Scale bar, 200 µm. Images are representative of at least three independent experiments. **B** Quantification of cleaved caspase-3-positive cells. Aβ42 significantly increases apoptotic cell numbers, and DEX treatment further enhances this response. **C** Quantification of PCNA-positive cells. Aβ42 induces a significant increase in proliferative activity, as shown by elevated PCNA-positive cell counts. DEX administration significantly reduces this proliferation, restoring values to near-control levels. In panels **B** and **C**, data are presented as mean ± standard deviation (SD) (*n* = 4 biological replicates per group). Statistical significance was determined using one-way analysis of variance (ANOVA). *p < 0.05, **p < 0.01, ****p < 0.0001

A disrupted balance between neuronal apoptosis and proliferation is a defining feature of AD. Cleaved caspase-3, a key executioner of apoptosis, is consistently elevated in AD brains and is widely recognized as a reliable marker of neuronal cell death [78–80]. To investigate the impact of DEX on Aβ-induced apoptosis, we performed immunofluorescent staining for cleaved caspase-3 in adult zebrafish brain sections. Aβ42 injection significantly increased cleaved caspase-3 expression compared to controls, indicating activation of apoptotic pathways (Fig. 4A). Interestingly, DEX treatment was associated with a further increase in the number of cleaved caspase-3-positive cells relative to the Aβ42-only group. Quantitative analysis confirmed this elevation, suggesting that DEX is associated with increased apoptotic signaling in response to Aβ toxicity (Fig. 4B). In parallel, we examined the expression of proliferating cell nuclear antigen (PCNA), a marker of DNA synthesis and cell cycle progression, often upregulated in zebrafish models of AD as part of a degeneration-driven regenerative response [81], [82]. As expected, PCNA expression was markedly increased in the Aβ42-injected group, consistent with a compensatory proliferative response to neurodegeneration (Fig. 4A) [50]. However, this increase was significantly attenuated following DEX treatment. Quantification of PCNA-positive cells revealed a significant reduction in the DEX-treated group, approaching control levels (Fig. 4C). This reduction may reflect decreased injury-induced compensatory proliferation rather than direct normalization of regenerative processes. Collectively, these findings indicate that DEX is associated with changes in apoptotic signaling and proliferation dynamics under Aβ stress conditions. Notably, the observed increase in cleaved caspase-3 occurs alongside reduced neuroinflammation, attenuated astrogliosis, and preservation of neuronal markers, although the functional implications of increased apoptosis remain unclear. These observations should be interpreted cautiously, as the underlying mechanisms were not directly investigated.

### DEX Modulates Glial Reactivity and Restores HuC/D Expression in the Zebrafish AD Model

**Fig. 5:**
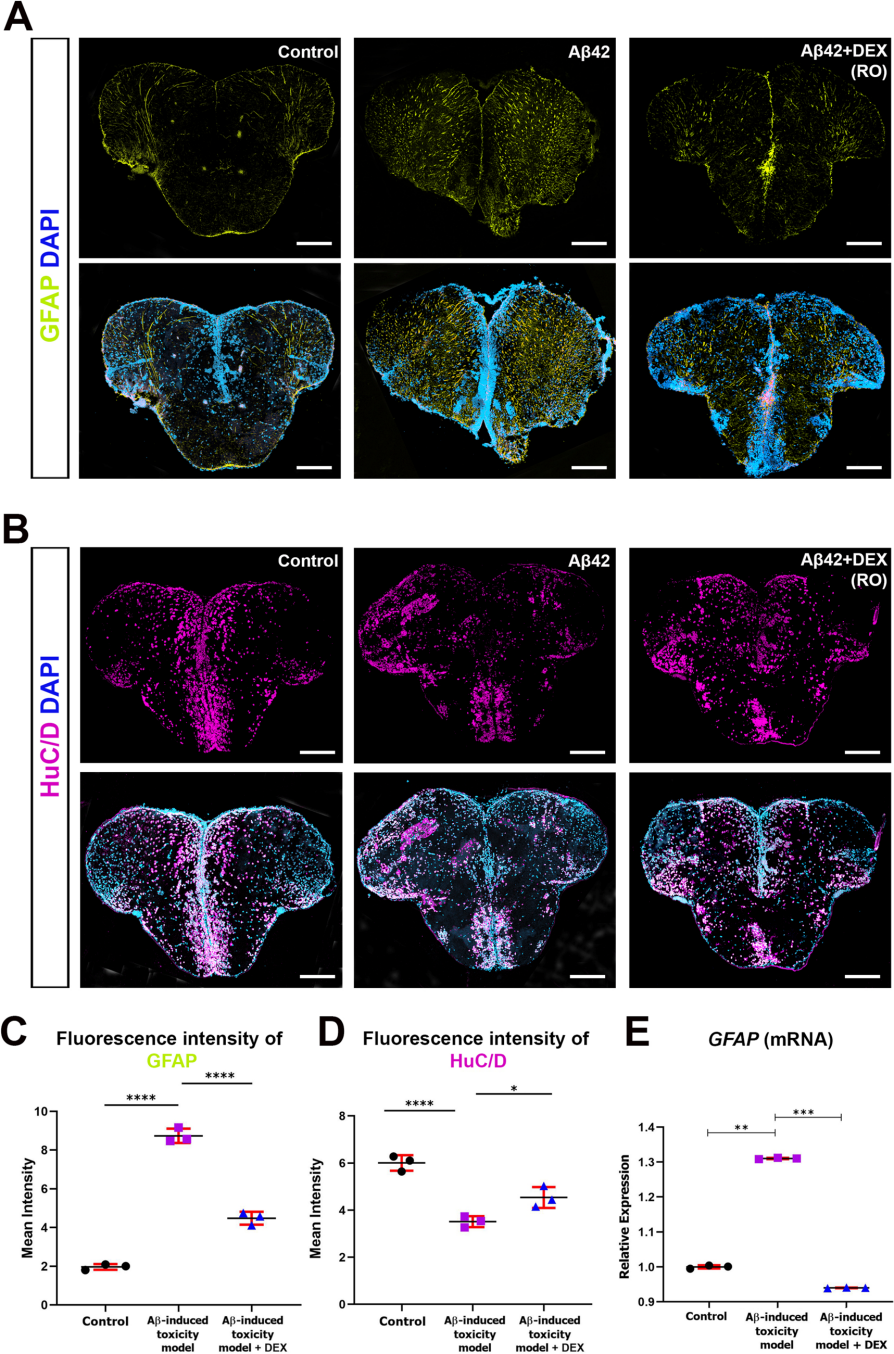
Glial and neuronal marker expression in the zebrafish AD model following DEX treatment. **A** Representative immunofluorescence images of zebrafish telencephalon sections stained for glial fibrillary acidic protein (GFAP; green; astrocyte marker) and 4′,6-diamidino-2-phenylindole (DAPI; blue; nuclei). Baseline GFAP expression is observed in control brains, while β-amyloid42 (Aβ42) administration induces robust glial activation, reflected by elevated GFAP levels. Dexmedetomidine (DEX) treatment markedly reduces GFAP signal, indicating suppression of reactive gliosis. **B** Representative immunofluorescence images of telencephalon sections stained for HuC/D (magenta; neuronal marker) and DAPI (blue; nuclei). Control brains exhibit widespread HuC/D expression, which is markedly reduced following Aβ42 exposure, indicating neuronal loss. DEX treatment restores HuC/D signal, suggesting preservation or recovery of neuronal populations. Scale bar, 200 µm. Images are representative of at least three independent experiments. **C** Quantification of GFAP fluorescence intensity presented as fold change relative to control. Aβ42 significantly increases GFAP signal, which is attenuated by DEX-treatment. **D** Quantification of HuC/D fluorescence intensity presented as fold change. Aβ42 administration results in a marked reduction in the intensity of the neuronal marker compared to the untreated control group. A pronounced elevation in HuC/D expression, while DEX treatment significantly restores neuronal marker intensity. **E** Relative mRNA expression of *GFAP* in whole-brain samples. Aβ42 significantly upregulates *GFAP* expression, whereas DEX treatment leads to significant downregulation, reducing expression to levels below control. In panels **C**–**E**, data are presented as mean ± standard deviation (SD) (*n* = 4 biological replicates per group in C-D; *n* = 3 biological replicates, each measured in triplicate in E). Statistical significance was determined using one-way analysis of variance (ANOVA). *p < 0.05, **p < 0.01, ***p < 0.001, ****p < 0.0001

Reactive astrogliosis is a hallmark of AD, typically characterized by astrocyte hypertrophy and increased expression of glial fibrillary acidic protein (GFAP). In response to Aβ accumulation and neurodegenerative stress, astrocytes become activated and express elevated levels of GFAP [83–85]. To evaluate astroglial activation in the Aβ42-induced zebrafish model and the effects of DEX on astroglial reactivity, we performed immunofluorescent staining for GFAP. Aβ42-injected zebrafish displayed widespread astrocyte activation throughout the brain, particularly in the telencephalon, compared to the control (Fig. 5A). Notably, GFAP expression was markedly reduced in the DEX-treated group. Quantitative analysis confirmed a significant decrease in GFAP fluorescence intensity following DEX treatment, indicating effective suppression of reactive astrogliosis (Fig. 5C). Consistent with these findings, qRT-PCR analysis revealed that *GFAP* mRNA expression was significantly elevated following Aβ42 injection and was reduced upon DEX treatment (Fig. 5E), supporting the observed changes at the transcriptional level. To assess neuronal integrity, we examined the expression of HuC/D, an RNA-binding protein selectively expressed in post-mitotic, differentiated neurons and commonly used as a marker of neuronal identity and integrity [86]. In AD models, reduced HuC/D expression is associated with neuronal loss and compromised neuronal health [87, 88]. In our model, Aβ42 injection led to a pronounced reduction in HuC/D expression, consistent with significant neuronal damage (Fig. 5B). In contrast, DEX treatment increased HuC/D expression toward control levels, suggesting preservation of neuronal marker expression through neuroprotective mechanisms. Quantification of HuC/D-positive cells supported these observations, revealing a significant decline in the Aβ42 group and a robust recovery in the DEX-treated group (Fig. 5D). GFAP and HuC/D levels in the Control + DEX group were not significantly different from controls, indicating that DEX does not affect basal astroglial or neuronal status under non-pathological conditions (Figure S1C-D). Collectively, these findings show that DEX attenuates Aβ42-induced glial activation and reverses the reduction in HuC/D expression, highlighting its potential protective role in reducing neuroinflammatory responses and modulating neurodegenerative processes in the zebrafish model of AD.

### DEX Ameliorates Aβ-Induced Behavioral Deficits in Zebrafish

**Fig. 6:**
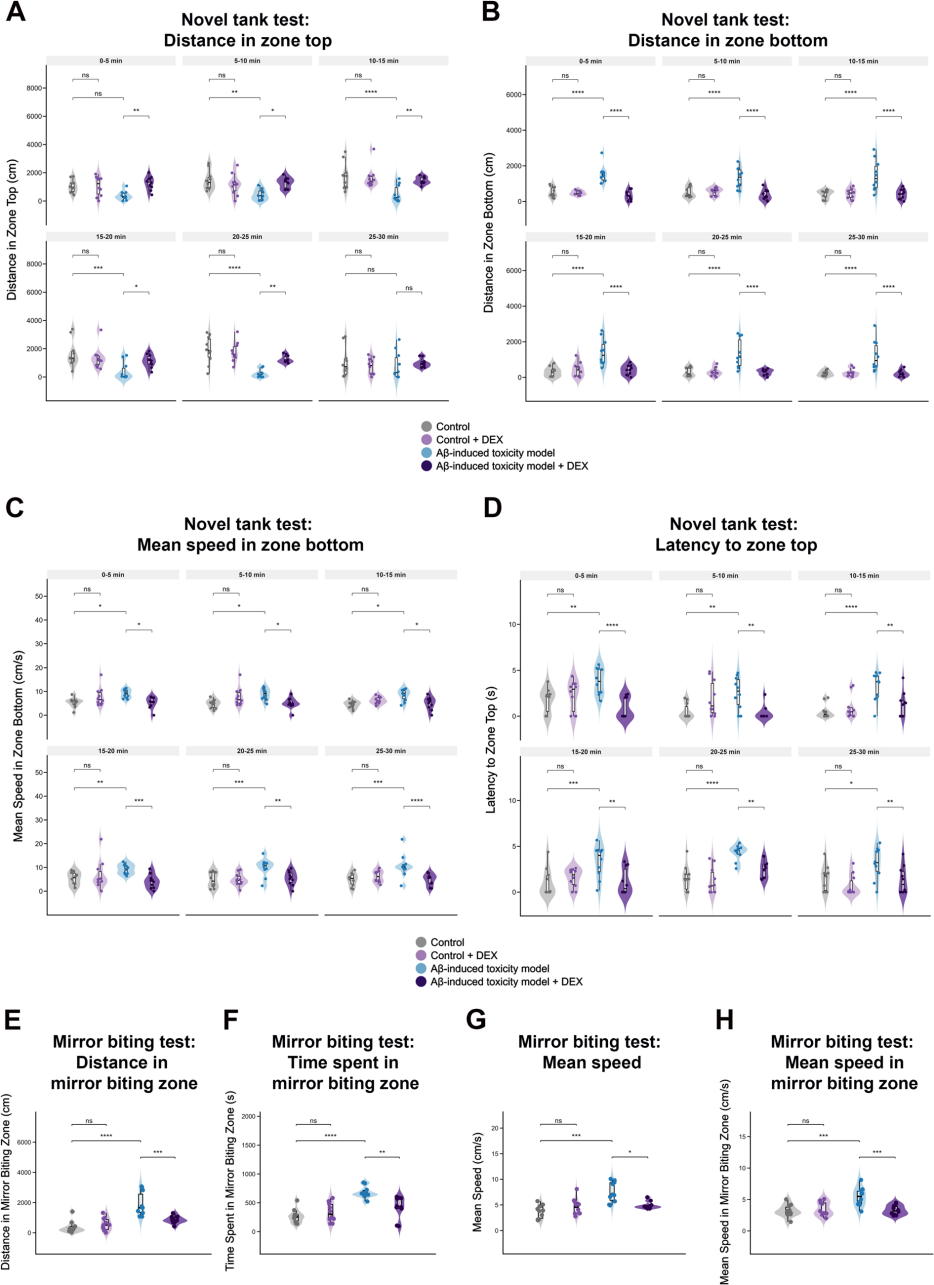
Behavioral outcomes in Aβ-treated zebrafish following DEX treatment. **A**–**D** Novel tank test: Behavioral responses were evaluated over 30 min using four metrics. **A** Distance traveled in the top zone. **B** Distance traveled in the bottom zone. **C** Mean swimming speed in the bottom zone. **D** Latency to enter the top zone. Control zebrafish exhibit normal exploratory behavior, characterized by frequent entries into and prolonged occupancy of the top zone. β-Amyloid42 (Aβ42) exposure induces anxiety-like responses, as evidenced by reduced top-zone activity and increased bottom-dwelling behavior. Dexmedetomidine (DEX) treatment significantly reverses these effects, restoring exploratory patterns. **E**–**H** Mirror biting test: aggression-like behaviors were assessed by measuring **E** distance traveled in the mirror biting zone, **F** time spent in the mirror biting zone, **G** total mean swimming speed, and **H** mean swimming speed within the mirror biting zone. Aβ42 administration leads to increased aggression-like behavior and hyperactivity, reflected by elevated locomotor activity and interaction with the mirror. DEX treatment significantly reduces these behavioral indices, indicating effective suppression of both hyperactivity and aggression-like responses. Data are presented as violin plots with median and interquartile range (IQR), with individual data points shown (*n* = 10 individual fish per group). Statistical analyses were performed using linear mixed-effects models for repeated measures (novel tank test) and one-way analysis of variance (ANOVA) or Welch’s ANOVA for non-repeated data (mirror biting test). Post hoc comparisons were conducted using multiple comparison procedures with adjusted *p*-values. For visualization purposes, latency to enter the top zone was log-transformed. *p < 0.05, **p < 0.01, ***p < 0.001, ****p < 0.0001; ns, not significant

Neurodegeneration in AD is frequently associated with anxiety-like behavior and altered social responses, such as increased aggression. To evaluate these behavioral changes in our zebrafish model of Aβ42-induced neurotoxicity, we employed validated protocols for anxiety and aggression assessment [54]. In the novel tank test, zebrafish typically display bottom-dwelling behavior in response to an unfamiliar environment, a well-established indicator of anxiety [66]. As they acclimate, their exploratory activity increases, and they begin occupying the upper regions of the tank. Zebrafish injected with Aβ42 exhibited pronounced anxiety-like behavior, characterized by a significant reduction in the distance traveled in the top zone, and an increase in the distance traveled in the bottom zone (Fig. 6A-B). Additionally, mean speed in bottom zone significantly increased (Fig. 6C), while latency to top zone was elevated compared to controls (Fig. 6D), confirming impaired exploratory behavior and heightened anxiety. Treatment with DEX reversed these anxiety-related alterations. DEX-treated fish showed increased distance traveled in the top zone and decreased distance in the bottom zone, indicating restored exploratory drive (Fig. 6A, B). Mean speed in bottom zone and latency to top zone were also normalized, or even improved, relative to controls, particularly during the later stages of the 30-min trial. DEX administration in the absence of Aβ42 (Control + DEX group) did not result in significant differences across measured behavioral parameters compared to controls, suggesting that the observed behavioral effects are unlikely to be attributable to non-specific sedative or locomotor effects of DEX, but rather reflect its modulatory action under Aβ-induced pathological conditions. These results support an anxiolytic effect of DEX and its capacity to reestablish behavioral homeostasis under amyloidogenic stress.

Following anxiety assessment, we conducted the mirror biting test to evaluate aggression-like behaviors. Aβ42-injected zebrafish demonstrated hyperactive and aggressive responses, as indicated by increased distance traveled (Fig. 6E), greater time spent in the mirror zone (Fig. 6F), and elevated mean swimming speed (Fig. 6G, H). In contrast, DEX treatment significantly reduced all these parameters, suggesting a normalization of aggressive behavior and restoration of social reactivity. DEX-treated significantly reduced all these parameters, suggesting a suppression of aberrant aggression and restoration of normal social behavior. Importantly, given the well-established sedative properties of DEX, its potential to confound behavioral outcomes independent of pathology was systematically evaluated through the inclusion of a Control + DEX group. No significant differences were detected between this group and controls across all behavioral parameters, indicating that DEX does not exert measurable effects on behavior under normal conditions (Fig. 6A–H). Together, these findings suggest that DEX mitigates Aβ42-induced anxiety- and aggression-like behaviors, supporting its role in modulating behavioral alterations in a zebrafish model of AD.

## Discussion

AD is a progressive neurodegenerative disorder characterized by irreversible cognitive decline, neuronal loss, and widespread neuroinflammation, posing a significant burden on patients, caregivers, and healthcare systems worldwide [89]. Despite decades of research, effective disease-modifying treatments remain elusive. Given this urgent need, repurposing clinically approved agents with known neuroprotective properties presents a promising strategy. DEX, an α2-adrenergic receptor agonist widely used in clinical settings for its sedative and analgesic properties, has recently gained attention for its anti-inflammatory anti-apoptotic and neuroprotective effects [90] Sanders, [91]. In the present study, we investigated the therapeutic potential of DEX in a zebrafish model of AD induced by CVMI of Aβ42. Our findings suggest that DEX exerts multi-level effects on key pathological features of the model, including Aβ accumulation, neuroinflammation, and behavioral alterations. Rather than indicating direct mechanistic actions, these results point to a coordinated modulation of disease-associated processes under Aβ-induced conditions.

### Dose Optimization and Safety Profile of DEX in Zebrafish

In line with findings from mammalian studies, our results demonstrate that DEX exerts dose-dependent effects in the zebrafish model, emphasizing the importance of precise dose optimization. While 250 µM DEX was well-tolerated in zebrafish larvae, causing no observable developmental abnormalities or mortality, exposure to a higher concentration of 500 µM led to significant morphological defects, including spinal curvature and edema, and resulted in 40% mortality. This biphasic response is consistent with previous reports describing DEX-induced toxicity at elevated doses. In rodent models of endotoxin-induced sepsis, DEX has been shown to exert anti-inflammatory effects by suppressing pro-inflammatory cytokine production and reducing microglial activation [92]. However, high-dose DEX exposure has also been associated with adverse physiological outcomes such as cardiorespiratory depression, cellular stress, hypertension, and increased mortality [71, 72, 93, 94]. This narrow therapeutic window may be partly explained by the pharmacokinetic properties of DEX, including rapid systemic distribution, a short elimination half-life of 2–3 h and high lipophilicity that facilitates efficient blood–brain barrier penetration [95, 96]. Taken together, these data suggest that while DEX is associated with beneficial and anti-inflammatory effects, it also possesses a relatively narrow therapeutic window in both vertebrate and in vitro systems. Therefore, careful titration is essential to maximize its therapeutic potential while minimizing toxicity in experimental and translational contexts.

### DEX Reduces Aβ Load and Normalizes Gene Expression

Aβ accumulation is a central hallmark of AD pathogenesis, initiating a cascade of neurotoxic events that include chronic inflammation, oxidative stress, and tau hyperphosphorylation. Aβ activates glial cells, particularly microglia and astrocytes, triggering the release of pro-inflammatory cytokines such as IL-1β and TNF-α, which further exacerbate neuronal damage [97]. Moreover, Aβ42 enhances the transcription of *BACE1* via activation of the JNK/c-Jun signaling pathway, establishing a positive feedback loop that perpetuates Aβ production and neuronal dysfunction [98]. Concurrently, Aβ impairs mitochondrial function and increases ROS, fostering an oxidative environment that further upregulates *BACE1* and other genes involved in amyloidogenesis [99, 100]. In parallel, Aβ-induced activation of kinases such as GSK-3β leads to tau hyperphosphorylation, microtubule destabilization, and synaptic loss, amplifying neurodegeneration and reinforcing Aβ accumulation [101, 102]. Several preclinical studies in AD models have demonstrated that DEX can attenuate these pathological processes. For instance, DEX reduces ferroptosis in hippocampal neurons and alleviates neuronal atrophy, as indicated by the restoration of Nissl bodies [103]. In 5XFAD transgenic mice, DEX prevented Aβ deposition and improved cognitive function [104]. Additionally, in isoflurane-exposed aged rats, DEX suppressed amyloidogenic APP processing and increased the expression of CREB and BDNF, which are critical regulators of synaptic plasticity and memory [105]. Consistent with these observations, our zebrafish model of Aβ42 toxicity revealed that DEX significantly reduced Aβ burden and downregulated key genes involved in the amyloidogenic pathway. These effects suggest that DEX may mitigate Aβ pathology through multi-level effects, potentially involving alteration of Aβ-related gene expression as well as reduction of Aβ accumulation. An important consideration is whether the observed transcriptional changes reflect direct pharmacological effects of DEX or secondary consequences of reduced Aβ burden. Given that DEX treatment significantly attenuated Aβ accumulation in our model, the normalization of gene expression may, at least in part, represent an indirect response to reduced neurotoxicity. Together, these findings suggest that the observed effects may arise from a combination of primary drug actions and downstream secondary responses. Further studies will be required to disentangle these mechanisms.

### DEX Attenuates Neuroinflammation

Neuroinflammation is a key driver in the onset and progression of AD. Pathological triggers such as Aβ accumulation and tau hyperphosphorylation activate glial cells, particularly microglia and astrocytes, resulting in the release of pro-inflammatory cytokines including IL-1β, IL-6, and TNF-α [106, 107]. These inflammatory mediators disrupt neuron-glia communication, impair synaptic function, and contribute to neuronal death [15]. Persistent neuroinflammation also compromises the blood–brain barrier, enabling infiltration of peripheral immune cells and amplifying neuronal damage [108]. Notably, emerging evidence suggests that neuroinflammation may represent an early and possibly initiating event in AD pathogenesis, rather than a secondary response [109].

DEX has emerged as a modulator of neuroinflammation, with demonstrated effects across multiple models of neurodegeneration and neuroinflammatory disease [110–113]. Previous studies in mammalian systems have shown that DEX reduces pro-inflammatory cytokines such as TNF-α, IL-1β, and IL-6 and is associated with changes in microglial inflammatory states, potentially through pathways including TLR4/NF-κB and MAPK/ERK signaling [114–116]. In line with these observations, our zebrafish model of Aβ42-induced neuroinflammation revealed that DEX significantly suppressed the expression of L-plastin, a marker associated with activated immune cells, including microglia [117]. This was accompanied by reduced transcription of pro-inflammatory cytokines (*il6st*, *tnfa*, *il1b*) and a concomitant upregulation of the anti-inflammatory cytokine *il10*, reflecting changes in inflammatory gene expression toward a less inflammatory profile. However, the specific signaling pathways underlying these effects were not directly investigated in the present study and should therefore be interpreted in the context of existing literature.

### DEX Is Associated with Changes in Proliferation and Apoptotic Signaling

In zebrafish, exposure to injury stimuli such as Aβ42 triggers a strong injury-induced proliferative response, marked by the upregulation of PCNA, a key indicator of DNA synthesis and cell cycle progression. This proliferation reflects injury-induced gliogenesis and is commonly associated with neuroinflammation and tissue repair processes [50, 118]. While such responses are part of the injury response, excessive or dysregulated proliferation can lead to maladaptive remodeling and impede functional recovery. DEX has recently been shown to exert both anti-inflammatory and anti-proliferative effects across various models. In tumor systems, DEX suppresses cell proliferation by downregulating c-Myc, inhibiting mTOR signaling, and reducing the expression of metabolic regulators such as PCK2 [119]. It also inhibits tumor growth, invasion, and migration, while promoting apoptosis through miR-130a upregulation and suppression of EGR1 in hepatocellular carcinoma cells [120]. Consistent with these findings, our study demonstrated that DEX treatment significantly reduced Aβ-induced PCNA expression in the zebrafish brain, indicating modulation of injury-induced proliferative responses under Aβ stress conditions.

While DEX is typically regarded as anti-apoptotic in models of ischemia, trauma, and neurotoxicity [111, 112, 121–126], our findings suggest a more nuanced, context-dependent role. Specifically, the increased levels of cleaved caspase-3 observed in the DEX-treated group following Aβ42 exposure indicate increased apoptotic signaling; however, whether this reflects detrimental cell loss or regulated cellular turnover remains unclear. Notably, this increase occurs alongside reduced neuroinflammation, attenuated astrogliosis, and restoration of neuronal markers such as HuC/D. These concurrent observations may suggest preservation of overall tissue integrity, although causality cannot be inferred. This is particularly relevant in the adult zebrafish brain, which exhibits a strong injury-induced cellular response. The observed changes in apoptosis and proliferation may reflect a coordinated response to injury. In this context, DEX may modulate cellular responses to injury rather than directly enhancing regeneration under Aβ-induced stress conditions.

One potential mechanism underlying this controlled apoptotic response could involve autophagy-related pathways. Autophagy is a fundamental cellular process responsible for the removal of damaged organelles and aggregated proteins and is increasingly recognized as interacting with apoptotic signaling pathways [127, 128]. Previous studies have demonstrated that DEX can modulate autophagic activity in various disease models [129–131]. For example, DEX has been reported to enhance autophagy and promote the removal of damaged mitochondria, thereby reducing oxidative stress and apoptosis [132, 133]. In addition, DEX-mediated neuroprotection in Aβ-induced models has been associated with improvements in autophagy–lysosomal function [134]. In this context, the concurrent reduction in aberrant proliferation and increase in cleaved caspase-3 levels observed in our study may be consistent with a coordinated cellular response contributing to the maintenance of tissue homeostasis. However, as autophagy markers were not directly assessed, this interpretation requires further investigation.

### DEX Reduces Astrogliosis and Preserves Neuronal Marker Expression

Chronic activation of astrocytes contributes to glial scar formation, synaptic dysfunction, and the amplification of neuroinflammation through the sustained release of cytokines and chemokines [135]. Multiple studies have demonstrated that DEX can suppress astrocyte reactivity in various CNS injury models [132–137]. For example, in a traumatic brain injury model, DEX reduced GFAP expression and astrocytic reactivity while enhancing connexin 43 expression via activation of the PI3K-Akt-GSK-3β signaling pathway [138]. Similarly, in a model of chronic constriction injury, DEX attenuated neuropathic pain by downregulating HMGB1-mediated astrocyte activation and inhibiting GFAP expression via the TLR4/NF-κB pathway [132, 133, 139–141].

By limiting astrocyte-mediated inflammation, DEX helps reduce the neurotoxic microenvironment and may support neuronal integrity. Previous studies have shown that DEX inhibits activation of the NLRP3 inflammasome, thereby reducing glial reactivity and preventing neuronal apoptosis in diverse models of neuroinflammation [142], [143], [144–146]. In line with these neuroprotective effects, our data revealed a significant increase in HuC/D-positive cells in the zebrafish brain following DEX treatment. Aβ42 administration substantially decreased HuC/D expression, reflecting neuronal loss driven by Aβ-induced toxicity and inflammation. Remarkably, DEX treatment increased HuC/D levels toward control levels, consistent with preservation of neuronal marker expression.

Given the intrinsic regenerative capacity of the zebrafish CNS, the anti-inflammatory effects of DEX may contribute to a permissive environment for neurogenic processes. Together, these findings suggest that DEX is associated with attenuation of astrocyte-mediated inflammatory responses and preservation of neuronal marker expression. This potential effect underscores its relevance as a therapeutic candidate for protecting neuronal integrity in AD and other neurodegenerative conditions.

### DEX Mitigates Aβ42-Induced Anxiety- and Aggression-Like Behaviors

Beyond its molecular and cellular effects, DEX has demonstrated significant therapeutic benefits at the behavioral level in various models of neurodegeneration and neuroinflammation. For example, in the 5xFAD transgenic mouse model of AD, DEX administration improved spatial learning and memory performance in both the Morris water maze and Y-maze tests [104]. These improvements were associated with reduced Aβ deposition and decreased neuroinflammation via α2-adrenergic receptor-mediated mechanisms. Similarly, in scopolamine-induced models of cognitive impairment, DEX enhanced learning ability by modulating the BDNF/TrkB/CREB signaling axis, underscoring its role in supporting synaptic plasticity and cognitive function [147]. Beyond cognition, DEX has been shown to alleviate anxiety-like behavior and cognitive deficits in stress-induced models, in part by preserving gut microbiota composition, suggesting a role for the gut-brain axis in its neuroprotective effects [148]. Consistent with these findings, our zebrafish model revealed that DEX administration effectively counteracted Aβ42-induced behavioral deficits. In the novel tank test, DEX-treated animals exhibited increased exploratory behavior and reduced anxiety-like responses, indicative of improved adaptation to an unfamiliar environment and decreased stress sensitivity. In the mirror biting assay, DEX administration restored locomotor activity and normalized aggression-like behaviors that were dysregulated by Aβ42 exposure. Taken together, these results suggest that DEX not only ameliorates the cellular and molecular pathology induced by Aβ42 but also improves neurobehavioral outcomes. Its ability to attenuate anxiety- and aggression-like behaviors further underscores its promise as a multifaceted therapeutic candidate for AD and other neurodegenerative conditions.

### Limitations and Future Directions

The zebrafish model provides a powerful platform for investigating neurodegenerative processes, offering advantages such as rapid experimental assessment, genetic tractability, and in vivo imaging capabilities [149–152]. However, the zebrafish model captures only part of the structural and functional complexity of the human brain, and the acute Aβ42 injection approach used in this study reflects limited aspects of the chronic and progressive nature of AD. As such, the extent to which the observed effects translate to long-term neurodegenerative processes remains to be further validated in more advanced and chronic models.

In addition to these model-related limitations, several biological and mechanistic considerations should be noted. While the Aβ42 injection-based model enables rapid induction of Aβ burden and associated neuroinflammatory responses, this model represents only selected features of AD pathogenesis. Key components such as tau pathology, progressive synaptic dysfunction, and age-related changes were not directly addressed within the scope of this study. Similarly, the cellular and molecular mechanisms underlying the observed effects of DEX were not directly investigated. DEX has been shown to modulate neuroinflammatory pathways, including suppression of microglial activation and pro-inflammatory signaling [142, 153–155] and may additionally influence Aβ pathology through complementary mechanisms such as reduction of oxidative stress and potential enhancement of microglia-mediated Aβ clearance. Nevertheless, whether the observed transcriptional changes reflect direct pharmacological actions of DEX or secondary responses to reduced Aβ burden remains unclear. In addition, cytokine-related findings were based on mRNA expression levels, and protein-level validation was not performed.

RO administration appeared to be more effective than immersion in reducing Aβ plaque burden; however, the absence of direct pharmacokinetic measurements represents an important limitation. Specifically, the lack of quantitative data on brain DEX concentrations or uptake efficiency restricts precise evaluation of central drug exposure and limits interpretation of route-dependent differences. This limitation should be considered in the context of existing literature, where most pharmacological studies in zebrafish rely on functional and phenotypic outcomes rather than direct concentration measurements [156–159]. DEX exhibits a well-characterized pharmacokinetic profile, including rapid systemic distribution, high lipophilicity, and efficient blood–brain barrier penetration, supporting its potential to exert central effects [29, 160–162]. In this context, injection-based delivery methods such as RO administration are likely to provide more controlled and reproducible systemic exposure, whereas immersion is associated with variability in absorption and uptake. However, without direct pharmacokinetic data, the extent to which route-dependent effects are driven by differences in central drug availability remains uncertain.

Beyond pharmacokinetic considerations, dose selection also requires careful interpretation, as interspecies differences in metabolism, distribution, and drug exposure limit direct extrapolation to humans. DEX is clinically used as a sedative agent and is characterized by a relatively narrow therapeutic window, with higher doses associated with adverse effects such as cardiorespiratory suppression [163, 164]. These properties may also confound behavioral outcomes. In this context, the dose-dependent effects observed in our study are consistent with the known pharmacological profile of DEX, although their safety and efficacy will need to be further evaluated in mammalian systems. Nevertheless, the present findings provide important insight into the pharmacodynamic actions of DEX in modulating neuroinflammatory and Aβ-related processes, supporting its potential for further translational investigation.

In addition, variability and reproducibility should be interpreted with caution when considering the present findings. Although consistent effects were observed across multiple biological levels, including molecular, histological, and behavioral analyses, the relatively small sample sizes used in this study may limit the ability to fully capture inter-individual variability. In particular, molecular assays conducted with limited biological replicates may not fully capture the extent of biological variability within the population. Sample size was partly constrained by ethical considerations related to animal use. While efforts were made to ensure experimental consistency and minimize technical variability, further studies incorporating larger cohorts and independent replication experiments will be necessary to confirm the robustness and reproducibility of these findings.

In line with these limitations, future studies integrating pathway-specific approaches, cell-type-resolved analyses, pharmacokinetic and pharmacodynamic measurements, optimized dose ranges, and cross-species validation will be essential to further clarify the mechanisms underlying DEX action and to strengthen the translational relevance of these findings .

## Conclusion

This study demonstrates that DEX is associated with multi-level changes in a zebrafish model of Aβ42-induced pathology, including alterations in Aβ accumulation, neuroinflammatory responses, glial activation, and neuronal marker expression. Moreover, DEX was also associated with changes in apoptotic signaling and proliferation dynamics, suggesting context-dependent effects under Aβ-induced stress conditions. Behavioral analyses further indicated improvements in Aβ42-induced behavioral phenotypes.

Taken together, these findings suggest that DEX may influence multiple disease-relevant processes in this model. Given its clinical availability and well-characterized pharmacological profile, DEX may represent a candidate for drug repositioning in neurodegenerative disease contexts. However, these findings are based on an acute zebrafish model and should be interpreted with caution.

## Supplementary Information

Below is the link to the electronic supplementary material.

ESM 1 (DOCX 816 KB)

ESM 2 (DOCX 16.6 KB)

ESM 3 (DOCX 16.1 KB)

ESM 4 (DOCX 46.8 KB)

ESM 5 (DOCX 3.26 MB)

## Data Availability

The data supporting the findings of this study are available from the corresponding authors, L.I. and G.O., upon reasonable request.
