# Supplementary material for: Dexmedetomidine Exerts Multi-level Effects to Ameliorate Alzheimer’s Disease Pathology in the Adult Zebrafish Brain
Source: Mol Neurobiol. 2026 May 5;63(1):609. doi: 10.1007/s12035-026-05906-9 (PMC13139303; doi:10.1007/s12035-026-05906-9)
Supplement: Supplementary file 1 — (DOCX 816 KB) [file 12035_2026_5906_MOESM1_ESM.docx]

**Nazli et al. Supplementary Figures**


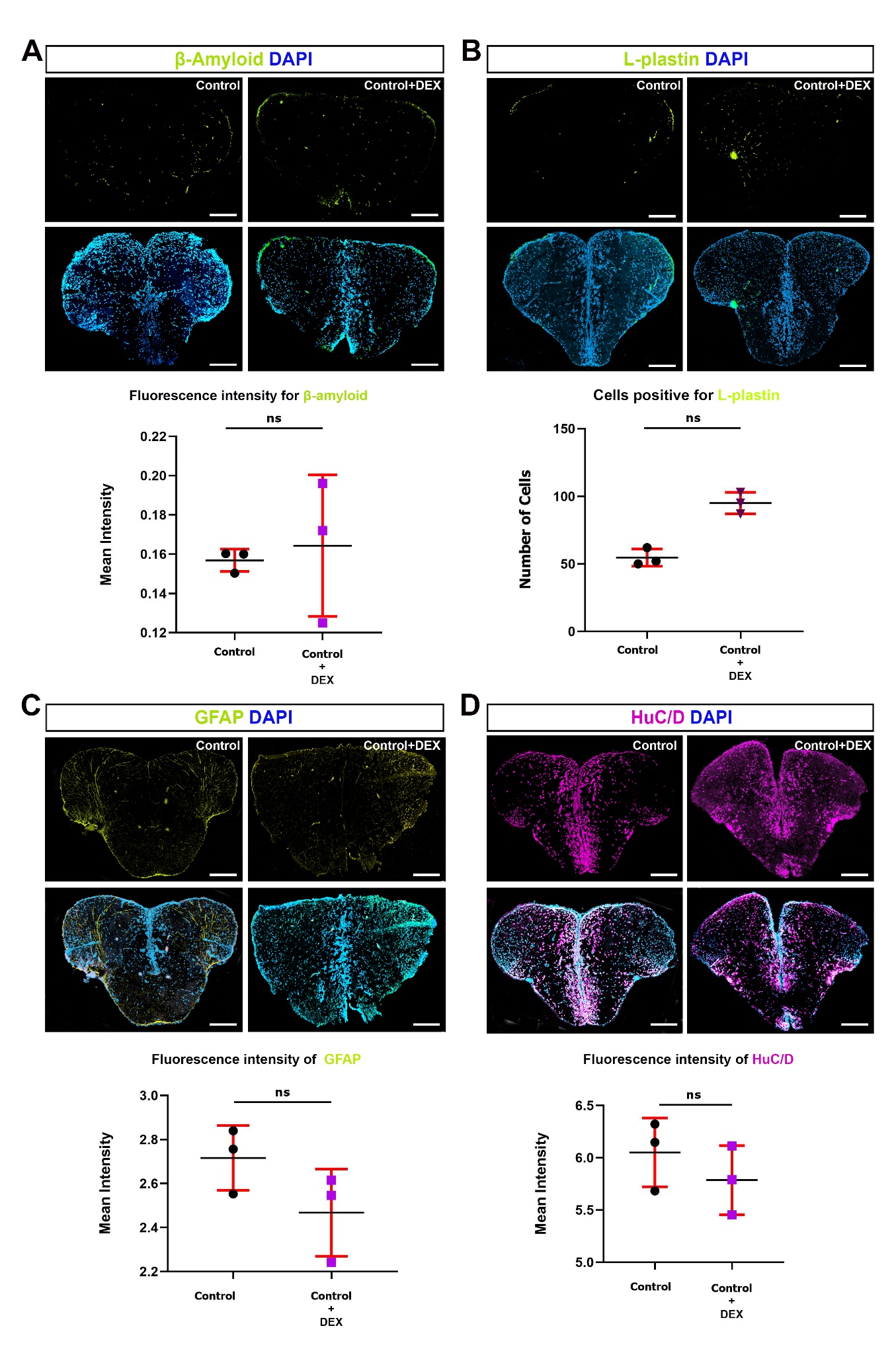


**Figure S1: Baseline pathological and cellular features in control and Control+DEX groups.** Representative immunofluorescence images of zebrafish telencephalon sections stained for (A) β-amyloid (green), (B) L-plastin (green; activated microglia), (C) glial fibrillary acidic protein (GFAP; green; astrocytes), and (D) HuC/D (magenta; neurons), with 4′,6-diamidino-2-phenylindole (DAPI; blue) as a nuclear counterstain. Sections are from control and control + dexmedetomidine (DEX) (retro-orbital injection) groups. Scale bar: 200 μm. Images are representative of at least three independent experiments. Quantification of β-amyloid fluorescence intensity (A), L-plastin-positive cell number (B), and fluorescence intensity of GFAP (C) and HuC/D (D) showed no statistically significant differences between control and control + DEX groups, indicating that DEX does not alter basal amyloid deposition, microglial activation, astroglial response, or neuronal integrity under non-pathological conditions. Data are presented as mean ± standard deviation (SD) (n = 3). Statistical significance was determined using an unpaired t-test. ns: not significant.


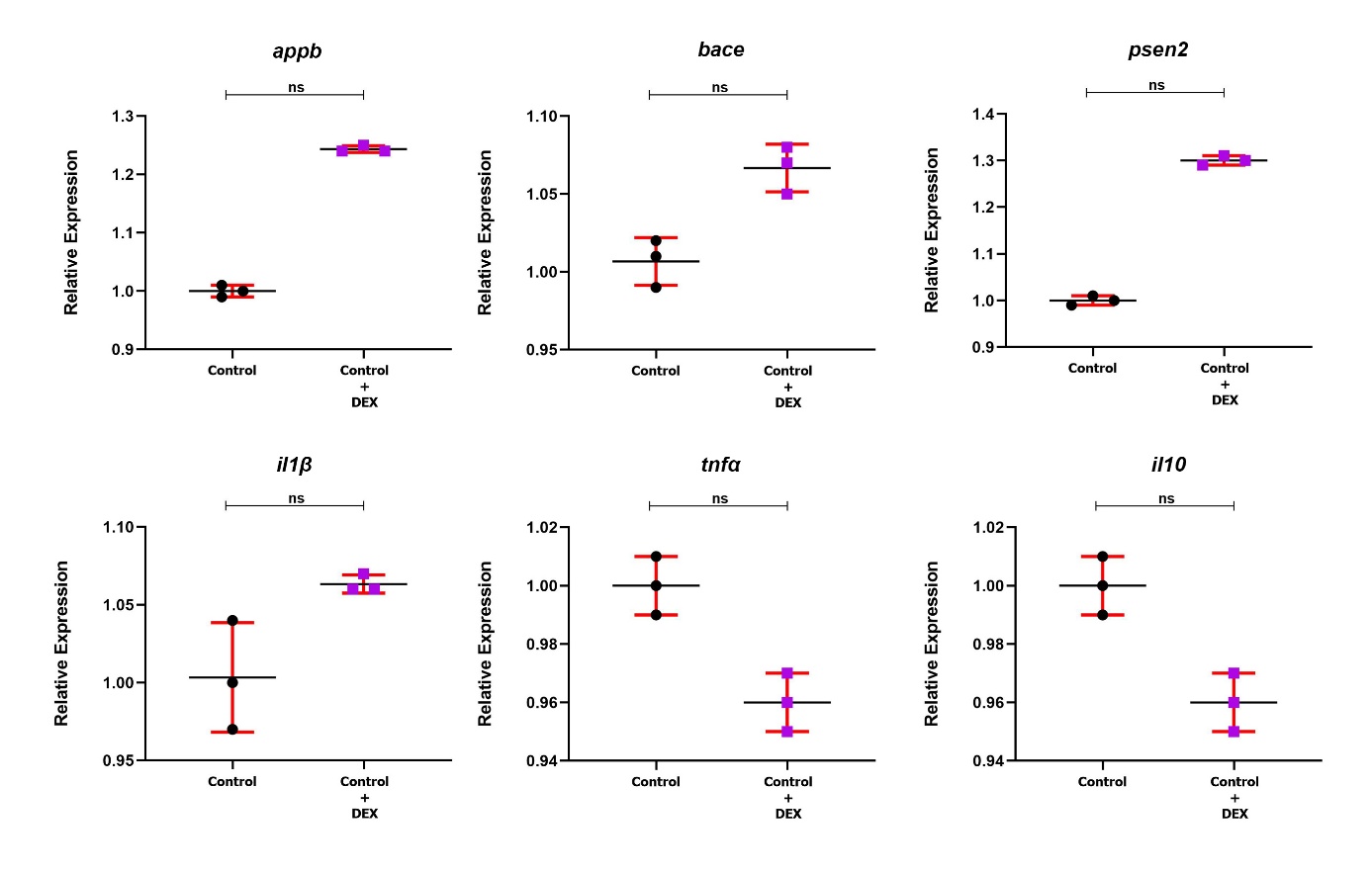


**Figure S2: Baseline gene expression profiles in control and Control+DEX groups.**  Relative mRNA expression levels of Alzheimer disease-related genes (*appb*, *bace*, and *psen2*) and inflammatory markers (*il1β*, *tnfα*, and *il10*) in whole-brain samples from control and control + dexmedetomidine (DEX) groups. DEX treatment did not result in statistically significant changes in the expression of any of these genes. Data are presented as mean ± standard deviation (SD) (n = 3). Statistical significance was determined using an unpaired t-test. ns: not significant.
