# Supplementary material for: Dexmedetomidine Exerts Multi-level Effects to Ameliorate Alzheimer’s Disease Pathology in the Adult Zebrafish Brain
Source: Mol Neurobiol. 2026 May 5;63(1):609. doi: 10.1007/s12035-026-05906-9 (PMC13139303; doi:10.1007/s12035-026-05906-9)
Supplement: Supplementary file 2 — (DOCX 16.6 KB) [file 12035_2026_5906_MOESM2_ESM.docx]

**Table S1: Forward and reverse primers used in qRT-PCR**

| **Target genes** | **5' to 3' Sequence** |
| --- | --- |
| zebrafish *appa* forward | CGAGAACGAACACGCTCACT |
| zebrafish *appa* reverse | CGAGAACGAACACGCTCACT |
| zebrafish *appb* forward | TCGATGAGCAAGACACCAGT |
| zebrafish *appb* reverse | CTGGAGCTTCCAGGTAACGA |
| zebrafish *psen1* forward | GGCTCATCCTCGCTGCTATT |
| zebrafish *psen1* reverse | TCCACAAGGATTCGCAGAGG |
| zebrafish *psen2* forward | GGCTCATCCTCGCTGCTATT |
| zebrafish *psen2* reverse | TCCACAAGGATTCGCAAGAGG |
| zebrafish *bace* forward | GGAGCCGGATACAACCACAA |
| zebrafish *bace* reverse | CATAGTACCACTCTCGGCGG |
| zebrafish *il1β* forward | GCTGGAGATCCAAACGGATA |
| zebrafish *il1β* reverse | ATACGCGGTGCTGATAAACC |
| zebrafish *il6st* forward | GGTCAGAACGGTCAGACGAA |
| zebrafish *il6st* reverse | ATGAAGAGCCGTGCCATAGT |
| zebrafish *il10* forward | CGACAGCACAGGAAATTTAGCAA |
| zebrafish *il10* reverse | AAGAAGCGTGAGCAGAGCAG |
| zebrafish *tnf* *α* forward | GCGCTTTTCTGAATCCTACG |
| zebrafish *tnf* *α* reverse | TGCCCAGTCTGTCTCCTTCT |
| zebrafish *rpl13a* forward | TCTGGAGGACTGTAAGAGGTATGC |
| zebrafish *rpl13a* reverse | AGACGCACAATCTTGAGAGCAG |
| zebrafish *gfap* forward | ACCCGTGACGGAGAGATCAT |
| zebrafish *gfap* reverse | GCCAGTGTCTGAGCCTCATT |
