# Supplementary material for: Dexmedetomidine Exerts Multi-level Effects to Ameliorate Alzheimer’s Disease Pathology in the Adult Zebrafish Brain
Source: Mol Neurobiol. 2026 May 5;63(1):609. doi: 10.1007/s12035-026-05906-9 (PMC13139303; doi:10.1007/s12035-026-05906-9)
Supplement: Supplementary file 3 — (DOCX 16.1 KB) [file 12035_2026_5906_MOESM3_ESM.docx]

| NAME | RATIO | CAT NO | COMPANY NAME | RRID | https://www.cellsignal.com/products/8243/datasheet?images=1&protocol=0&size=A4 |
| --- | --- | --- | --- | --- | --- |
| Anti-β-Amyloid | 1:500 | D54D2 | Cell Signaling Technology | AB_2797642 | https://www.thermofisher.com/order/genome-database/dataSheetPdf?producttype=antibody&productsubtype=antibody_primary&productId=A-21271&version=Local |
| Anti-HuC/HuD | 1:500 | A-21271 | Thermo Fisher | AB_221448 | https://doc.abcam.com/datasheets/active/ab154474/en-us/gfap-antibody-zrf-1-astrocyte-marker-ab154474.pdf |
| Anti-GFAP antibody | 1:500 | ab154474 | Abcam | AB_2571552 | https://www.ptglab.com/products/L-Plastin-Antibody-55208-1-AP.htm?srsltid=AfmBOoqSkjb81kcM9pxUSZcRqYstkiWtJHTiacBLxym7Q2bvLpAov1dv |
| Anti-L-Plastin | 1:200 | 55208-1-AP | Proteintech | AB_2881288 | https://www.cellsignal.com/products/9664/datasheet?images=1&protocol=0&size=A4 |
| Anti-cleaved caspase 3 | 1:300 | 9664S | Cell Signaling Technology | AB_2070042 | https://www.agilent.com/store/en_US/Prod-M087901-2/M087901-2 |
| Anti-Proliferating Cell Nuclear Antigen | 1:500 | M087901-2 | Agilent | AB_2052330 | https://www.jacksonimmuno.com/catalog/products/711-025-152 |
| Rhodamine (TRITC) Donkey Anti-Rabbit | 1:500 | 711–025-152 | Jackson Immunoresearch Laboratories | AB_2340588 | https://www.jacksonimmuno.com/catalog/products/715-175-151 |
| Cy5 Donkey Anti-Mouse | 1:500 | 715-175-151 | Jackson Immunoresearch Laboratories | AB_2340820 | https://www.cellsignal.com/products/4083/datasheet?images=1&protocol=0&size=A4 |

**Table S2: Validation of primary antibody specificity**
