# Supplementary material for: Dexmedetomidine Exerts Multi-level Effects to Ameliorate Alzheimer’s Disease Pathology in the Adult Zebrafish Brain
Source: Mol Neurobiol. 2026 May 5;63(1):609. doi: 10.1007/s12035-026-05906-9 (PMC13139303; doi:10.1007/s12035-026-05906-9)
Supplement: Supplementary file 4 — (DOCX 46.8 KB) [file 12035_2026_5906_MOESM4_ESM.docx]

**Table S3. Summary of statistical analyses, including model outputs and pairwise comparisons with adjusted p-values.**

**Abbrev.  Amyloid as Aβ-induced toxicity model, DEX as Aβ-induced toxicity model + DEX, DEX in water as Aβ-induced toxicity model + DEX in system water**

**A. Novel Tank**

Distance in the top zone:

| **Linear mixed-effects model** | | Group: F(3, 36) = 12.88, p < 0.001; Time: F(5, 180) = 4.03, p = 0.0017; Group × Time: F(15, 180) = 2.14, p = 0.0099 |
| --- | --- | --- |
| **Time Interval** | **Experimental Groups** | **Results** |
| 0–5 min | Amyloid vs Control | estimate = -661.00, SE = 291.78, t = -2.27, p = 0.0725 |
| 0–5 min | Amyloid vs DEX | estimate = -900.34, SE = 291.78, t = -3.09, p = 0.0071 |
| 0–5 min | Control+DEX vs Control | estimate = 31.78, SE = 291.78, t = 0.11, p = 0.9994 |
| 5–10 min | Amyloid vs Control | estimate = -1008.23, SE = 291.78, t = -3.46, p = 0.0021 |
| 5–10 min | Amyloid vs DEX | estimate = -847.93, SE = 291.78, t = -2.91, p = 0.0124 |
| 5–10 min | Control+DEX vs Control | estimate = -298.52, SE = 291.78, t = -1.02, p = 0.6682 |
| 10–15 min | Amyloid vs Control | estimate = -1285.38, SE = 291.78, t = -4.41, p = 5.63 × 10⁻⁵ |
| 10–15 min | Amyloid vs DEX | estimate = -941.79, SE = 291.78, t = -3.23, p = 0.0045 |
| 10–15 min | Control+DEX vs Control | estimate = -146.57, SE = 291.78, t = -0.50, p = 0.9434 |
| 15–20 min | Amyloid vs Control | estimate = -1233.54, SE = 291.78, t = -4.23, p = 1.16 × 10⁻⁴ |
| 15–20 min | Amyloid vs DEX | estimate = -740.73, SE = 291.78, t = -2.54, p = 0.0357 |
| 15–20 min | Control+DEX vs Control | estimate = -297.79, SE = 291.78, t = -1.02, p = 0.6700 |
| 20–25 min | Amyloid vs Control | estimate = -1669.46, SE = 291.78, t = -5.72, p = 1.42 × 10⁻⁷ |
| 20–25 min | Amyloid vs DEX | estimate = -1020.14, SE = 291.78, t = -3.50, p = 0.0018 |
| 20–25 min | Control+DEX vs Control | estimate = -139.60, SE = 291.78, t = -0.48, p = 0.9506 |
| 25–30 min | Amyloid vs Control | estimate = -228.23, SE = 291.78, t = -0.78, p = 0.8198 |
| 25–30 min | Amyloid vs DEX | estimate = -187.86, SE = 291.78, t = -0.64, p = 0.8898 |
| 25–30 min | Control+DEX vs Control | estimate = -266.10, SE = 291.78, t = -0.91, p = 0.7416 |

Distance in the bottom zone:

| **Linear mixed-effects model** | | Group: F(3, 36) = 58.70, p < 0.001; Time: F(5, 180) = 2.05, p = 0.0743; Group × Time: F(15, 180) = 0.21, p = 0.9993 |
| --- | --- | --- |
| **Time Interval** | **Experimental Groups** | **Results** |
| 0–5 min | Amyloid vs Control | estimate = 1009.65, SE = 190.45, t = 5.30, p =8.72 × 10⁻⁷ |
| 0–5 min | Amyloid vs DEX | estimate = 1178.63, SE = 190.45, t = 6.19, p = 9.45 × 10⁻⁹ |
| 0–5 min | Control+DEX vs Control | estimate = -32.01, SE = 190.45, t = -0.17, p = 0.9976 |
| 5–10 min | Amyloid vs Control | estimate = 814.01, SE = 190.45, t = 4.27, p = 8.74 × 10⁻⁵ |
| 5–10 min | Amyloid vs DEX | estimate = 971.86, SE = 190.45, t = 5.10, p = 2.25 × 10⁻⁶ |
| 5–10 min | Control+DEX vs Control | estimate = 47.90, SE = 190.45, t = 0.25, p = 0.9922 |
| 10–15 min | Amyloid vs Control | estimate = 1037.39, SE = 190.45, t = 5.45, p = 4.29 × 10⁻⁷ |
| 10–15 min | Amyloid vs DEX | estimate = 1045.20, SE = 190.45, t = 5.49, p = 3.50 × 10⁻⁷ |
| 10–15 min | Control+DEX vs Control | estimate = 20.79, SE = 190.45, t = 0.11, p = 0.9993 |
| 15–20 min | Amyloid vs Control | estimate = 1085.39, SE = 190.45, t = 5.70, p = 1.22 × 10⁻⁷ |
| 15–20 min | Amyloid vs DEX | estimate = 1001.51, SE = 190.45, t = 5.26, p = 1.07 × 10⁻⁶ |
| 15–20 min | Control+DEX vs Control | estimate = 112.66, SE = 190.45, t = 0.59, p = 0.9118 |
| 20–25 min | Amyloid vs Control | estimate = 1021.41, SE = 190.45, t = 5.36, p = 6.47 × 10⁻⁷ |
| 20–25 min | Amyloid vs DEX | estimate = 1026.19, SE = 190.45, t = 5.39, p = 5.72 × 10⁻⁷ |
| 20–25 min | Control+DEX vs Control | estimate = 2.57, SE = 190.45, t = 0.01, p = 0.9999 |
| 25–30 min | Amyloid vs Control | estimate = 973.31, SE = 190.45, t = 5.11, p = 2.17 × 10⁻⁶ |
| 25–30 min | Amyloid vs DEX | estimate = 989.56, SE = 190.45, t = 5.20, p = 1.45 × 10⁻⁶ |
| 25–30 min | Control+DEX vs Control | estimate = 27.00, SE = 190.45, t = 0.14, p = 0.9986 |

Mean Speed in the bottom zone:

| **Linear mixed-effects model** | | Group: F(3, 36) = 9.98, p < 0.001; Time: F(5, 180) = 0.66, p = 0.6575; Group × Time: F(15, 180) = 1.10, p = 0.3602 |
| --- | --- | --- |
| **Time Interval** | **Experimental Groups** | **Results** |
| 0–5 min | Amyloid vs Control | estimate = 3.70, SE = 1.35, t = 2.74, p = 0.0211 |
| 0–5 min | Amyloid vs DEX | estimate = 3.56, SE = 1.35, t = 2.63, p = 0.0282 |
| 0–5 min | Control+DEX vs Control | estimate = 2.46, SE = 1.35, t = 1.82, p = 0.1975 |
| 5–10 min | Amyloid vs Control | estimate = 3.97, SE = 1.35, t = 2.94, p = 0.0118 |
| 5–10 min | Amyloid vs DEX | estimate = 3.77, SE = 1.35, t = 2.79, p = 0.0183 |
| 5–10 min | Control+DEX vs Control | estimate = 2.94, SE = 1.35, t = 2.18, p = 0.0914 |
| 10–15 min | Amyloid vs Control | estimate = 4.01, SE = 1.35, t = 2.97, p = 0.0108 |
| 10–15 min | Amyloid vs DEX | estimate = 3.47, SE = 1.35, t = 2.56, p = 0.0342 |
| 10–15 min | Control+DEX vs Control | estimate = 1.81, SE = 1.35, t = 1.34, p = 0.4527 |
| 15–20 min | Amyloid vs Control | estimate = 4.07, SE = 1.35, t = 3.01, p = 0.0095 |
| 15–20 min | Amyloid vs DEX | estimate = 5.26, SE = 1.35, t = 3.89, p = 4.78 × 10⁻⁴ |
| 15–20 min | Control+DEX vs Control | estimate = 1.61, SE = 1.35, t = 1.19, p = 0.5529 |
| 20–25 min | Amyloid vs Control | estimate = 5.17, SE = 1.35, t = 3.83, p = 6.10 × 10⁻⁴ |
| 20–25 min | Amyloid vs DEX | estimate = 4.47, SE = 1.35, t = 3.31, p = 0.0037 |
| 20–25 min | Control+DEX vs Control | estimate = 0.13, SE = 1.35, t = 0.09, p = 0.9996 |
| 25–30 min | Amyloid vs Control | estimate = 5.81, SE = 1.35, t = 4.30, p = 1.02 × 10⁻⁴ |
| 25–30 min | Amyloid vs DEX | estimate = 5.88, SE = 1.35, t = 4.35, p = 8.25 × 10⁻⁵ |
| 25–30 min | Control+DEX vs Control | estimate = 1.29, SE = 1.35, t = 0.96, p = 0.7129 |

Latency to enter the top zone:

| **Linear mixed-effects model** | | Group: F(3, 36) = 22.42, p < 0.001; Time: F(5, 180) = 4.05, p = 0.0017; Group × Time: F(15, 180) = 1.71, p = 0.0528 |
| --- | --- | --- |
| **Time Interval** | **Experimental Groups** | **Results** |
| 0–5 min | Amyloid vs Control | estimate = 2.01, SE = 0.61, t = 3.28, p = 0.0036 |
| 0–5 min | Amyloid vs DEX | estimate = 2.91, SE = 0.61, t = 4.74, p = 1.22 × 10⁻⁵ |
| 0–5 min | Control+DEX vs Control | estimate = 0.49, SE = 0.61, t = 0.80, p = 0.8111 |
| 5–10 min | Amyloid vs Control | estimate = 2.02, SE = 0.61, t = 3.28, p = 0.0036 |
| 5–10 min | Amyloid vs DEX | estimate = 2.21, SE = 0.61, t = 3.61, p = 0.0012 |
| 5–10 min | Control+DEX vs Control | estimate = 1.40, SE = 0.61, t = 2.28, p = 0.0701 |
| 10–15 min | Amyloid vs Control | estimate = 2.67, SE = 0.61, t = 4.35, p = 6.15 × 10⁻⁴ |
| 10–15 min | Amyloid vs DEX | estimate = 1.95, SE = 0.61, t = 3.17, p = 0.0052 |
| 10–15 min | Control+DEX vs Control | estimate = 0.40, SE = 0.61, t = 0.65, p = 0.8853 |
| 15–20 min | Amyloid vs Control | estimate = 2.32, SE = 0.61, t = 3.78, p = 0.0006 |
| 15–20 min | Amyloid vs DEX | estimate = 2.23, SE = 0.61, t = 3.63, p = 0.0011 |
| 15–20 min | Control+DEX vs Control | estimate = 0.51, SE = 0.61, t = 0.83, p = 0.7910 |
| 20–25 min | Amyloid vs Control | estimate = 2.80, SE = 0.61, t = 4.57, p = 2.58 × 10⁻⁵ |
| 20–25 min | Amyloid vs DEX | estimate = 1.85, SE = 0.61, t = 3.01, p = 0.0088 |
| 20–25 min | Control+DEX vs Control | estimate = -0.28, SE = 0.61, t = -0.46, p = 0.9554 |
| 25–30 min | Amyloid vs Control | estimate = 1.61, SE = 0.61, t = 2.63, p = 0.0275 |
| 25–30 min | Amyloid vs DEX | estimate = 1.86, SE = 0.61, t = 3.03, p = 0.0083 |
| 25–30 min | Control+DEX vs Control | estimate = -0.81, SE = 0.61, t = -1.32, p = 0.4623 |

**B. Mirror Biting**

Time spent in the mirror biting zone:

| **One-way ANOVA** | | F(3, 36) = 15.06, p = 1.64 × 10⁻⁶ |
| --- | --- | --- |
| **Time Interval** | **Experimental Groups** | **Results** |
| 0-15 min | Control+DEX vs Control | estimate = 64.73, SE = 65.87, t = 0.98, p = 0.7024 |
| 0-15 min | Amyloid vs Control | estimate = 411.68, SE = 65.87, t = 6.25, p = 9.69 × 10⁻⁷ |
| 0-15 min | Amyloid vs DEX | estimate = 257.23, SE = 65.87, t = 3.90, p = 0.0012 |

Distance in the mirror biting zone:

| **One-way ANOVA** | | F(3, 36) = 17.95, p = 2.72 × 10⁻⁷ |
| --- | --- | --- |
| **Time Interval** | **Experimental Groups** | **Results** |
| 0–15 min | Control+DEX vs Control | estimate = 190.69, SE = 225.27, t = 0.85, p = 0.7871 |
| 0–15 min | Amyloid vs Control | estimate = 1514.56, SE = 225.27, t = 6.72, p = 2.28 × 10⁻⁷ |
| 0–15 min | Amyloid vs DEX | estimate = 1032.83, SE = 225.27, t = 4.58, p = 1.59 × 10⁻⁴ |

Total mean speed:

| Welch’s ANOVA | | Welch’s ANOVA: F(3, 18.44) = 7.90, p = 0.0010 |
| --- | --- | --- |
| **Time Interval** | **Experimental Groups** | **Results** |
| 0–15 min | Control+DEX vs Control | estimate = 0.98, 95% CI [-0.89, 2.85], p = 0.4640 |
| 0–15 min | Amyloid vs Control | estimate = 3.78, 95% CI [1.62, 5.93], p = 0.000707 |
| 0–15 min | Amyloid vs DEX | estimate = -2.54, 95% CI [-4.54, -0.55], p = 0.0130 |

Mean speed in the mirror biting zone:

| one-way ANOVA | | F(3, 36) = 9.03, p = 0.000137 |
| --- | --- | --- |
| **Time Interval** | **Experimental Groups** | **Results** |
| 0–15 min | Control+DEX vs Control | estimate = 0.28, SE = 0.49, t = 0.57, p = 0.9211 |
| 0–15 min | Amyloid vs Control | estimate = 2.22, SE = 0.49, t = 4.48, p = 0.000217 |
| 0–15 min | Amyloid vs DEX | estimate = 2.12, SE = 0.49, t = 4.28, p = 0.000400 |

**C. Positive Cell Counts**

| one-way ANOVA  (Cleaved-caspase Marker) | | F(2, 6) = 48.47, p = 1.98 × 10⁻⁴ |
| --- | --- | --- |
| **Marker** | **Experimental Groups** | **Results** |
| Cleaved-caspase | Amyloid vs Control | estimate = 32.33, SE = 5.33, t = 6.06, p = 0.0018 |
| Cleaved-caspase | Amyloid vs DEX | estimate = -19.67, SE = 5.33, t = -3.69, p = 0.0204 |
| One-way ANOVA  (PCNA Marker) | | F(2, 6) = 69.56, p = 7.06 × 10⁻⁵ |
| **Marker** | **Experimental Groups** | **Results** |
| PCNA | Amyloid vs Control | estimate = 74.33, SE = 7.34, t = 10.12, p = 1.001 × 10⁻⁴ |
| PCNA | Amyloid vs DEX | estimate = 75.67, SE = 7.34, t = 10.30, p = 9.76 × 10⁻⁵ |
| One-way ANOVA  (L-plastin Marker) | | F(2, 6) = 61.01, p = 0.000103 |
| **Marker** | **Experimental Groups** | **Results** |
| L_plastin | Amyloid vs Control | estimate = 65.00, SE = 6.85, t = 9.49, p = 1.56 × 10⁻⁴ |
| L_plastin | Amyloid vs DEX | estimate = 66.00, SE = 6.85, t = 9.64, p = 1.43 × 10⁻⁴ |
| Unpaired two-tailed t-test | | |
| **Marker** | **Experimental Groups** | **Results** |
| L_plastin | Control_DEX vs Control | t(4) = -0.49, p = 0.6505 |

**D. Fluorescence intensity**

| **Marker** | **Analysis** | **Experimental Groups** | **Results** |
| --- | --- | --- | --- |
| ß-amyloid | one-way ANOVA | Control, Amyloid, DEX, DEX in system water | F(3, 8) = 115.16, p = 6.40 × 10⁻⁷ |
| ß-amyloid | one-way ANOVA | Amyloid vs Control | estimate = 1.86, SE = 0.11, t = 17.13, p = 5.50 × 10⁻⁷ |
| ß-amyloid | one-way ANOVA | Amyloid vs DEX | estimate = 1.60, SE = 0.11, t = 14.74, p = 1.76 × 10⁻⁶ |
| ß-amyloid | one-way ANOVA | Amyloid vs DEX in system water | estimate = 1.25, SE = 0.11, t = 11.50, p = 1.19 × 10⁻⁵ |
| ß-amyloid | unpaired t-test | Control_DEX vs Control | t(4) = -0.40, p = 0.7127 |
| GFAP | one-way ANOVA | Control, Amyloid, DEX | F(2, 6) = 391.67, p = 4.39 × 10⁻⁷ |
| GFAP | one-way ANOVA | Amyloid vs Control | estimate = 6.77, SE = 0.24, t = 27.68, p = 2.94 × 10⁻⁷ |
| GFAP | one-way ANOVA | Amyloid vs DEX | estimate = 4.26, SE = 0.24, t = 17.41, p = 4.61 × 10⁻⁶ |
| GFAP | unpaired t-test | Control_DEX vs Control | t(4) = 1.74, p = 0.1565 |
| HuC/D | one-way ANOVA | Control, Amyloid, DEX | F(2, 6) = 39.24, p = 3.58 × 10⁻⁴ |
| HuC/D | one-way ANOVA | Amyloid vs Control | estimate = -2.50, SE = 0.28, t = -8.81, p = 2.37 × 10⁻⁴ |
| HuC/D | one-way ANOVA | Amyloid vs DEX | estimate = -1.03, SE = 0.28, t = -3.63, p = 0.0218 |
| HuC/D | unpaired t-test | Control_DEX vs Control | t(4) = 0.98, p = 0.3807 |

**E. RT-qPCR**

| **Gene** | **Analysis** | **Experimental Groups** | **Results** |
| --- | --- | --- | --- |
| appa | one-way ANOVA | Control, Amyloid, DEX | F(2, 6) = 130.53, p = 1.13 × 10⁻⁵ |
| appa | one-way ANOVA | Amyloid vs Control | estimate = -1.09, SE = 0.12, t =-8.9, p = 2.17 × 10⁻⁵ |
| appa | one-way ANOVA | Amyloid vs DEX | estimate = 1.19, SE = 0.12, t =9.7, p = 1.32 × 10⁻⁵ |
| psen2 | one-way ANOVA | Control, Amyloid, DEX | F(2, 6) = 210.81, p = 2.76 × 10⁻⁶ |
| psen2 | one-way ANOVA | Amyloid vs Control | estimate = -0.88, SE = 0.08, t =-11.0, p = 5.97 × 10⁻⁶ |
| psen2 | one-way ANOVA | Amyloid vs DEX | estimate = 0.99, SE = 0.08, t =12.4, p = 2.99 × 10⁻⁶ |
| psen2 | unpaired t-test | Control_DEX vs Control | t(4) = -2.23, p = 0.08963 |
| il6st | one-way ANOVA | Control, Amyloid, DEX | F(2, 6) = 60.61, p = 1.05 × 10⁻⁴ |
| il6st | one-way ANOVA | Amyloid vs Control | estimate = -0.72, SE = 0.12, t =-6.0, p = 2.12 × 10⁻⁴ |
| il6st | one-way ANOVA | Amyloid vs DEX | estimate = 0.80, SE = 0.12, t =6.7, p = 1.16 × 10⁻⁴ |
| gfap | one-way ANOVA | Control, Amyloid, DEX | F(2, 6) = 39.48, p = 3.52 × 10⁻⁴ |
| gfap | one-way ANOVA | Amyloid vs Control | estimate = -0.31, SE = 0.07, t =-4.4, p = 9.30 × 10⁻⁴ |
| gfap | one-way ANOVA | Amyloid vs DEX | estimate = 0.37, SE = 0.07, t =5.2, p = 3.29 × 10⁻⁴ |
| bace | one-way ANOVA | Control, Amyloid, DEX | F(2, 6) = 246.67, p = 1.73 × 10⁻⁶ |
| bace | one-way ANOVA | Amyloid vs Control | estimate = -4.66, SE = 0.34, t =-13.7, p = 1.79 × 10⁻⁶ |
| bace | one-way ANOVA | Amyloid vs DEX | estimate = 4.05, SE = 0.34, t =11.9, p = 4.09 × 10⁻⁶ |
| bace | unpaired t-test | Control_DEX vs Control | t(4) = -1.17, p = 0.30780 |
| appb | one-way ANOVA | Control, Amyloid, DEX | F(2, 6) = 395.83, p = 4.26 × 10⁻⁷ |
| appb | one-way ANOVA | Amyloid vs Control | estimate = -1.21, SE = 0.08, t =-15.1, p = 1.26 × 10⁻⁶ |
| appb | one-way ANOVA | Amyloid vs DEX | estimate = 1.48, SE = 0.08, t =18.5, p = 3.92 × 10⁻⁷ |
| appb | unpaired t-test | Control_DEX vs Control | t(4) = -2.06, p = 0.10895 |
| psen1 | one-way ANOVA | Control, Amyloid, DEX | F(2, 6) = 38.80, p = 3.70 × 10⁻⁴ |
| psen1 | one-way ANOVA | Amyloid vs Control | estimate = -0.55, SE = 0.15, t =-3.7, p = 0.0025 |
| psen1 | one-way ANOVA | Amyloid vs DEX | estimate = 0.84, SE = 0.15, t =5.6, p = 2.60 × 10⁻⁴ |
| tnfα | one-way ANOVA | Control, Amyloid, DEX | F(2, 6) = 3.85 × 10²⁸, p < 2.2 × 10⁻¹⁶ |
| tnfα | one-way ANOVA | Amyloid vs Control | estimate = -0.34, SE =0.00, p < 2.2 × 10⁻¹⁶ |
| tnfα | one-way ANOVA | Amyloid vs DEX | estimate = 0.11, SE =0.00, p < 2.2 × 10⁻¹⁶ |
| tnfα | unpaired t-test | Control_DEX vs Control | t(4) = 0.25, p = 0.81613 |
| il10 | one-way ANOVA | Control, Amyloid, DEX | F(2, 6) = 2.10 × 10³², p < 2.2 × 10⁻¹⁶ |
| il10 | one-way ANOVA | Amyloid vs Control | estimate = -0.21, SE =0.00001, p < 2.2 × 10⁻¹⁶ |
| il10 | one-way ANOVA | Amyloid vs DEX | estimate = -1.19, SE =0.00001, p < 2.2 × 10⁻¹⁶ |
| il10 | unpaired t-test | Control_DEX vs Control | t(4) = 1.07, p = 0.34510 |
| il1ß | one-way ANOVA | Control, Amyloid, DEX | F(2, 6) = 32.23, p = 6.17 × 10⁻⁴ |
| il1ß | one-way ANOVA | Amyloid vs Control | estimate = -1.44, SE = 0.28, t =-5.1, p = 4.997 × 10⁻⁴ |
| il1ß | one-way ANOVA | Amyloid vs DEX | estimate = 1.08, SE = 0.28, t =3.8, p = 0.00230 |
| il1ß | unpaired t-test | Control_DEX vs Control | t(4) = 0.33, p = 0.75679 |
