# Supplementary material for: Dexmedetomidine Exerts Multi-level Effects to Ameliorate Alzheimer’s Disease Pathology in the Adult Zebrafish Brain
Source: Mol Neurobiol. 2026 May 5;63(1):609. doi: 10.1007/s12035-026-05906-9 (PMC13139303; doi:10.1007/s12035-026-05906-9)
Supplement: Supplementary file 5 — (DOCX 3.26 MB) [file 12035_2026_5906_MOESM5_ESM.docx]

**Table S4: Trajectory plot of all behavioral assays**

| **Novel Tank Assay : Control** | | |
| --- | --- | --- |
| 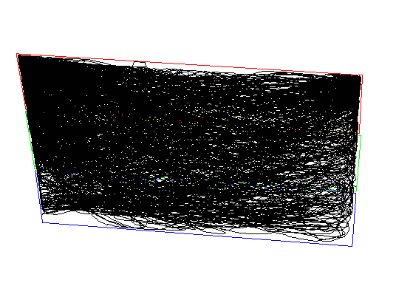 | 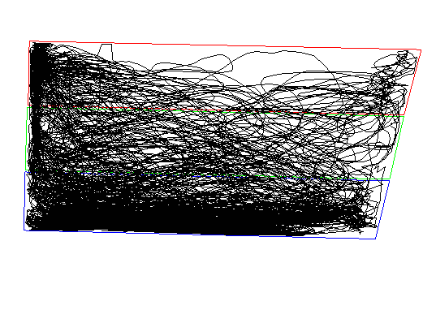 | 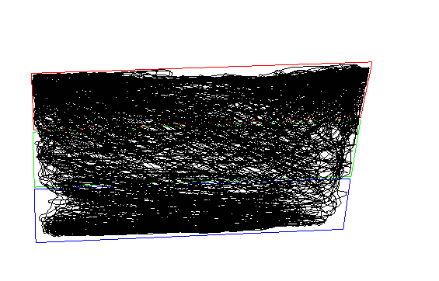 |
| 1 | 2 | 3 |
| 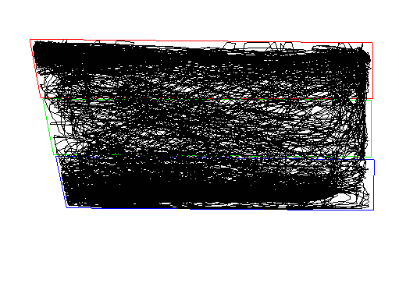 | 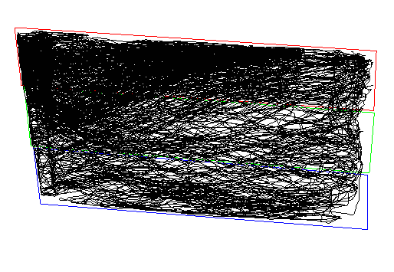 | 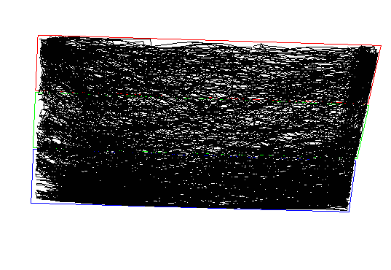 |
| 4 | 5 | 6 |
| 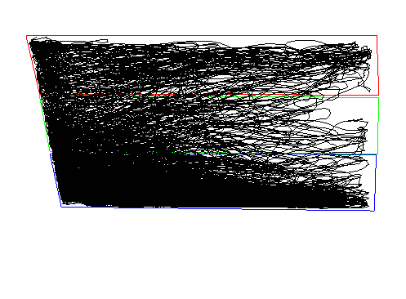 | 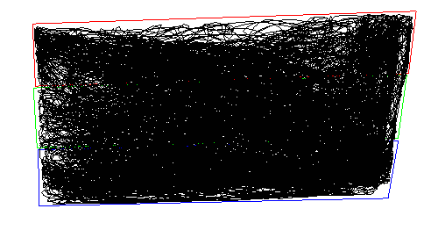 | 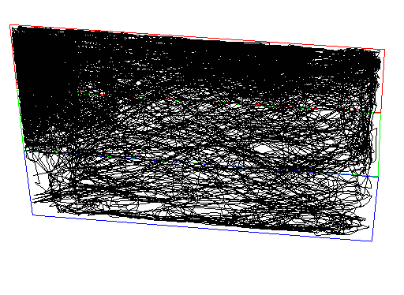 |
| 7 | 8 | 9 |
|  | 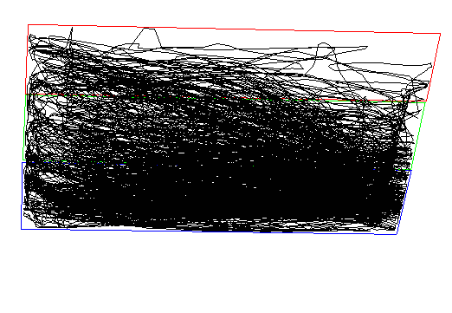 |  |
|  | 10 |  |
| **Novel Tank Assay : Aβ42 induced toxicity model** | | |
| 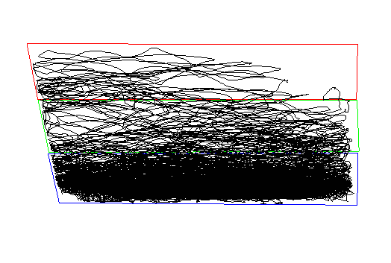 | 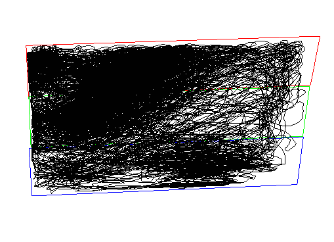 | 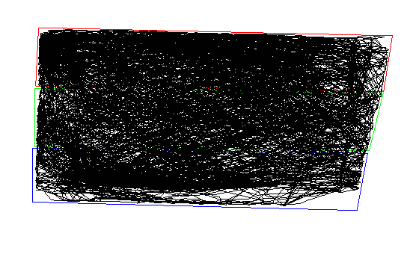 |
| 1 | 2 | 3 |
| 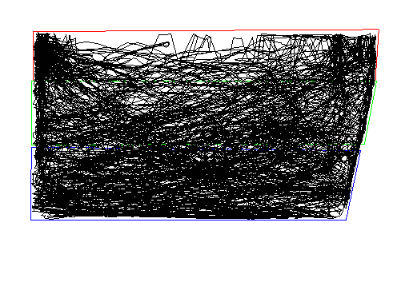 | 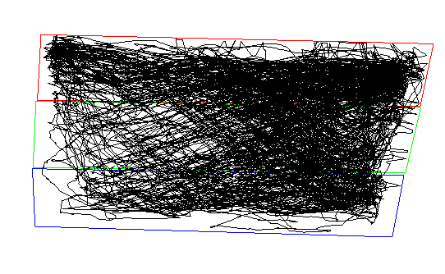 | 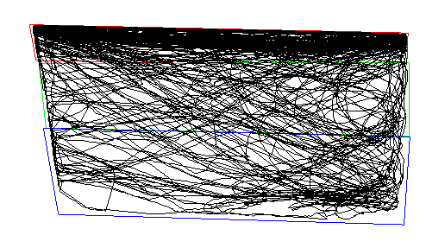 |
| 4 | 5 | 6 |
| 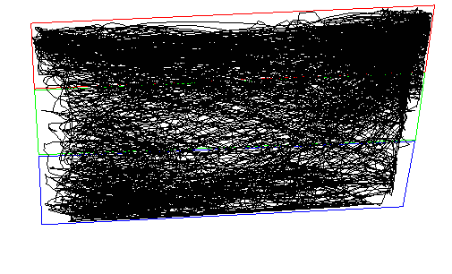 | 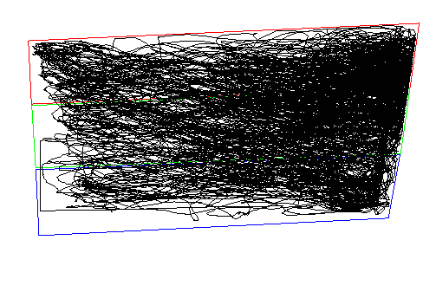 | 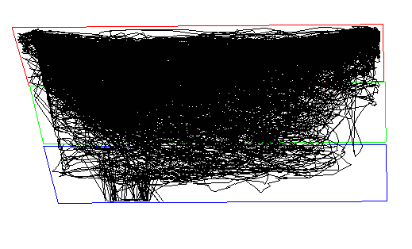 |
| 7 | 8 | 9 |
|  | 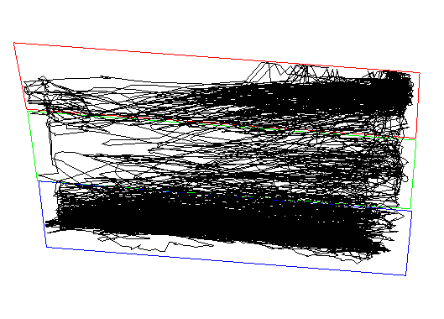 |  |
|  | 10 |  |
|  |  |  |
| **Novel Tank Assay : Aβ42-induced toxicity model + DEX** | | |
| 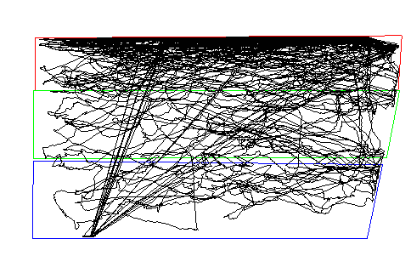 | 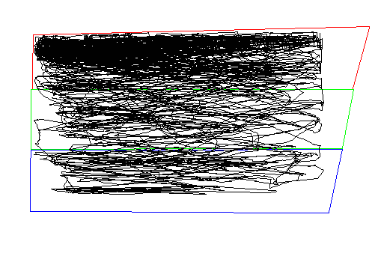 | 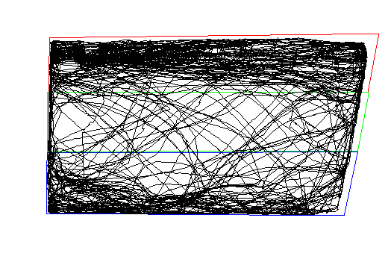 |
| 1 | 2 | 3 |
| 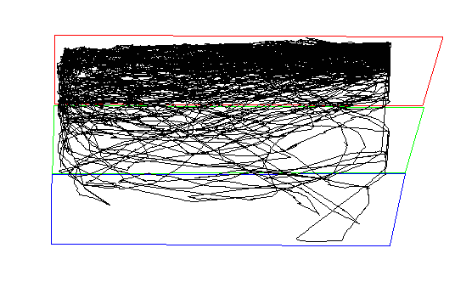 | 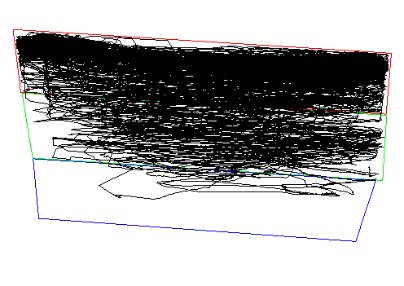 | 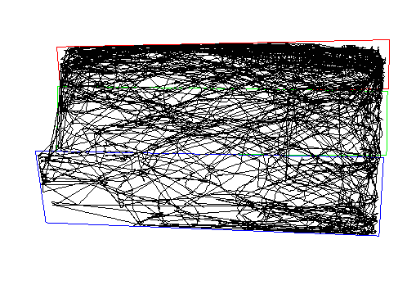 |
| 4 | 5 | 6 |
| 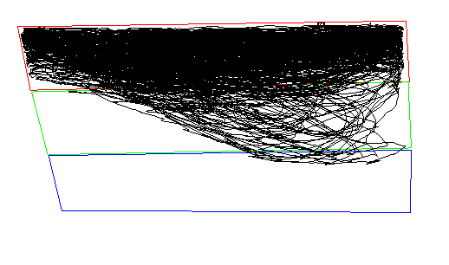 | 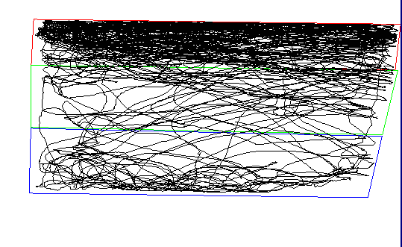 | 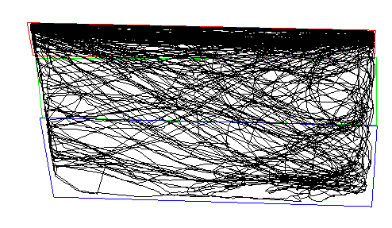 |
| 7 | 8 | 9 |
|  | 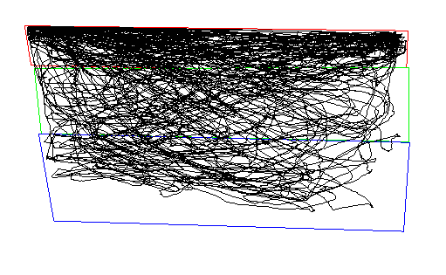 |  |
|  | 10 |  |

| **Novel Tank Assay : Control + DEX** | | |
| --- | --- | --- |
| 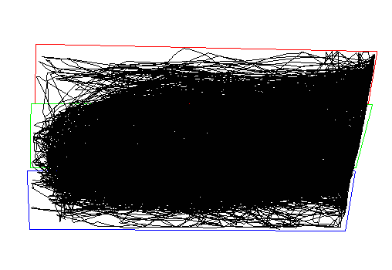 | 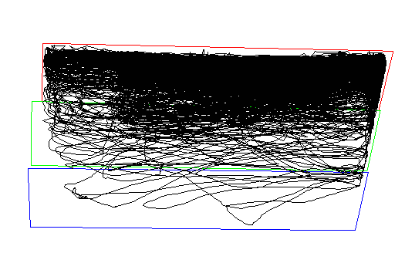 | 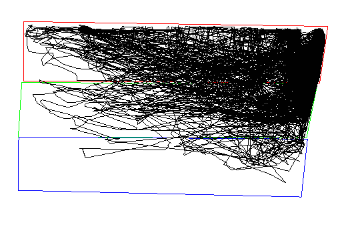 |
| 1 | 2 | 3 |
| 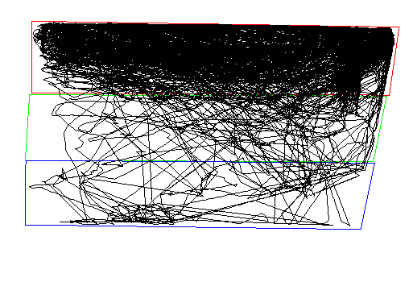 | 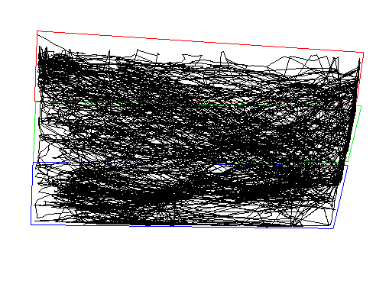 | 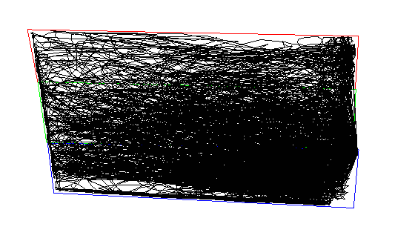 |
| 4 | 5 | 6 |
| 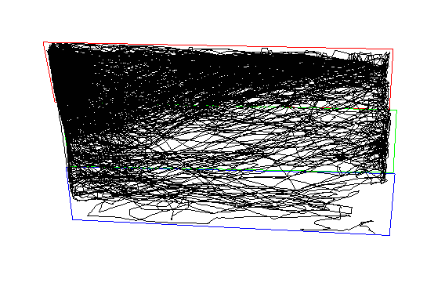 | 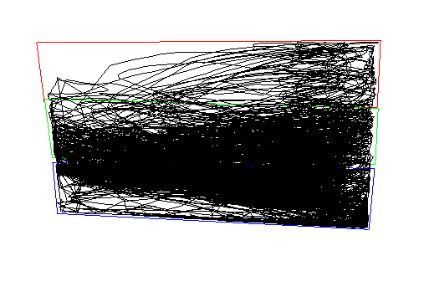 | 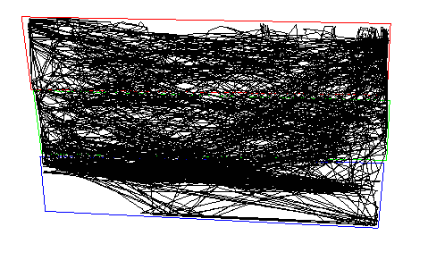 |
| 7 | 8 | 9 |
|  | 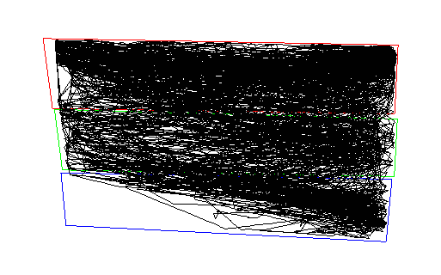 |  |
|  | 10 |  |
| **Mirror Biting Assay : Control** | | |
| 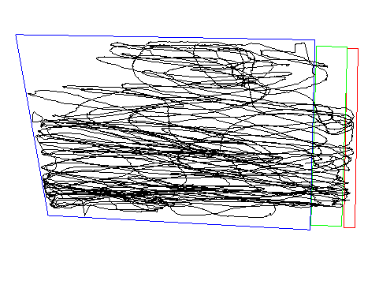 | 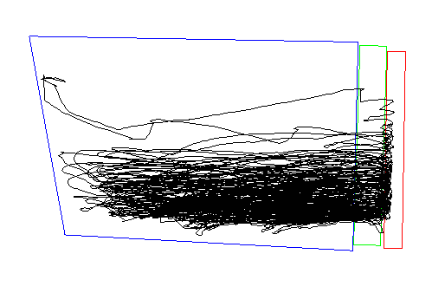 | 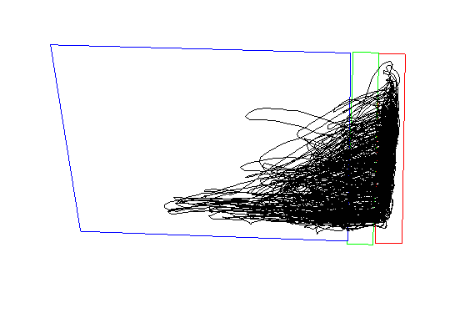 |
| 1 | 2 | 3 |
| 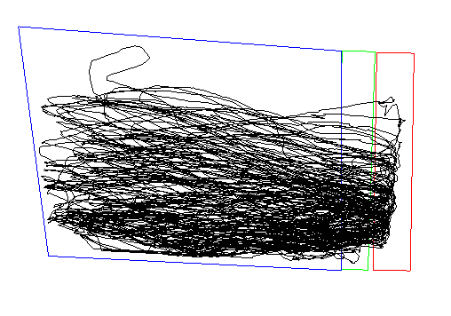 | 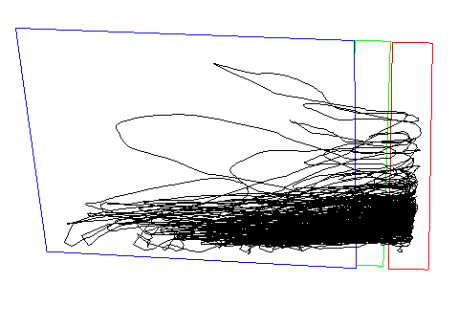 | 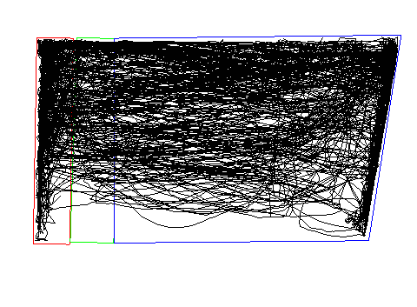 |
| 4 | 5 | 6 |
| 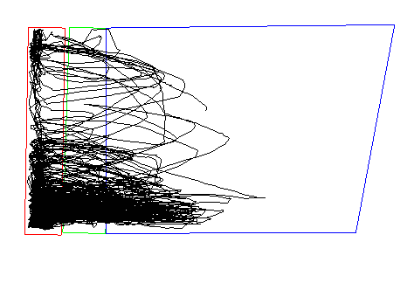 | 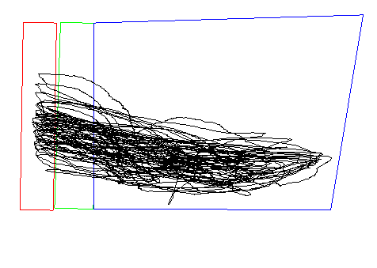 | 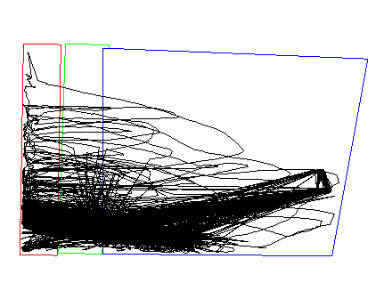 |
| 7 | 8 | 9 |
|  | 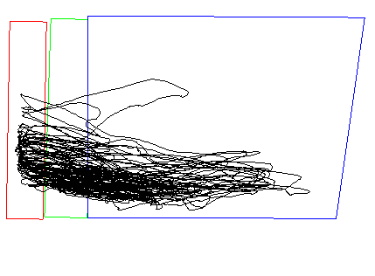 |  |
|  | 10 |  |
| **Mirror Biting Assay : Aβ42-induced toxicity model** | | |
| 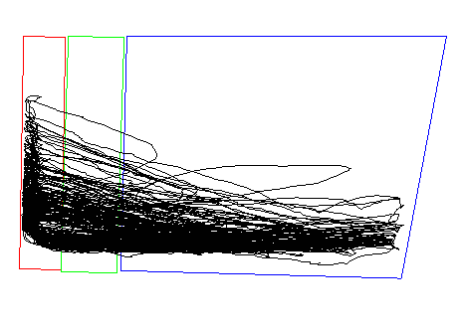 | 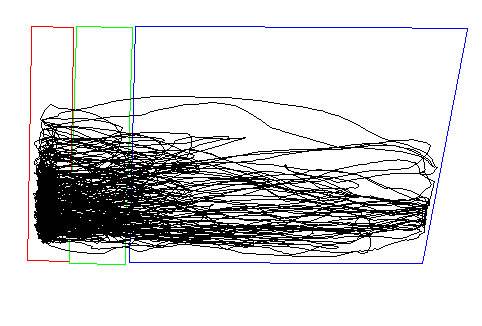 | 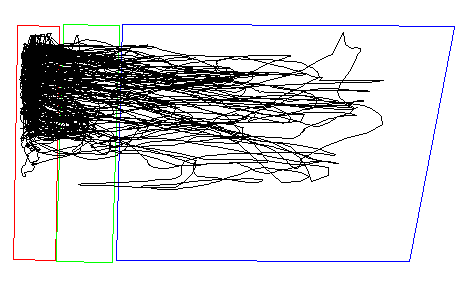 |
| 1 | 2 | 3 |
| 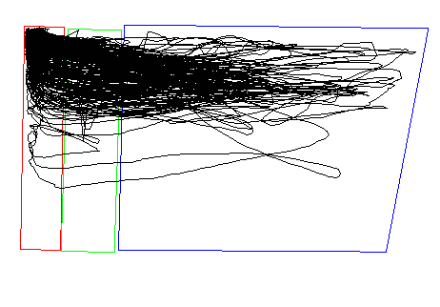 | 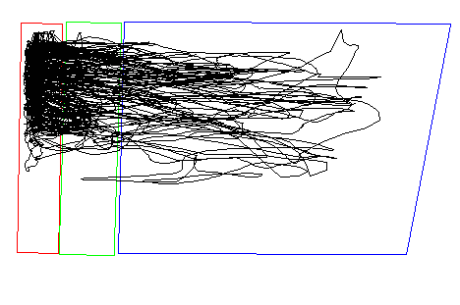 | 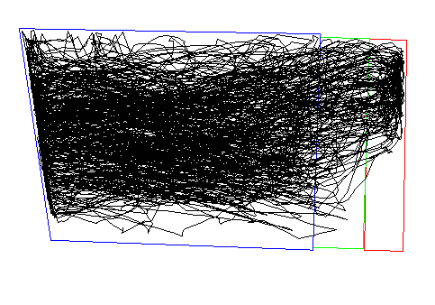 |
| 4 | 5 | 6 |
| 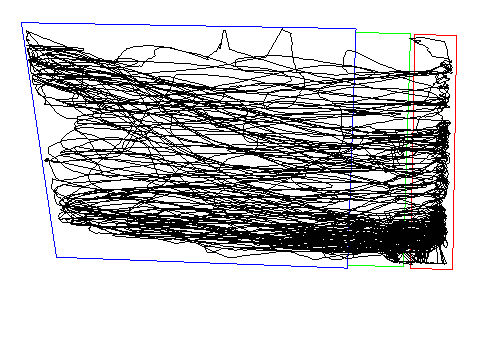 | 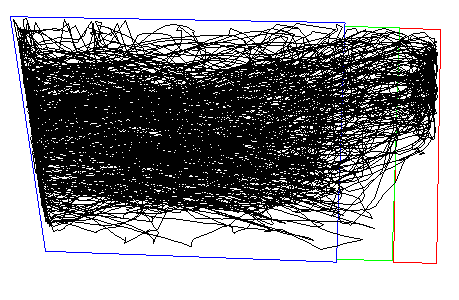 | 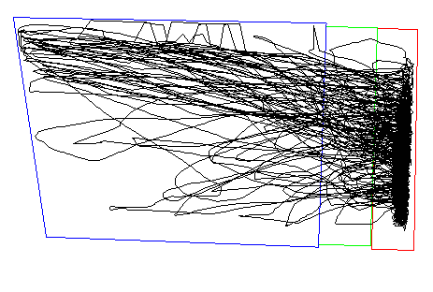 |
| 7 | 8 | 9 |
|  | 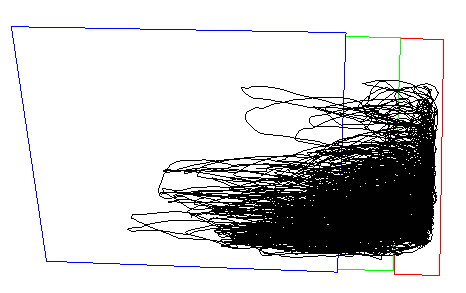 |  |
|  | 10 |  |
| **Mirror Biting Assay : Aβ42-induced toxicity model + DEX** | | |
| 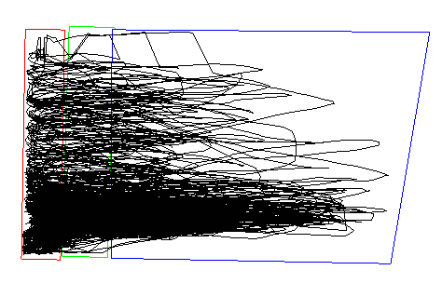 | 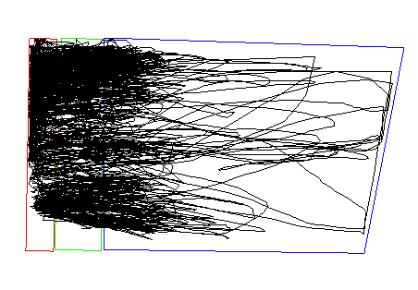 | 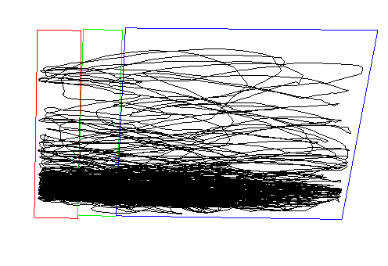 |
| 1 | 2 | 3 |
| 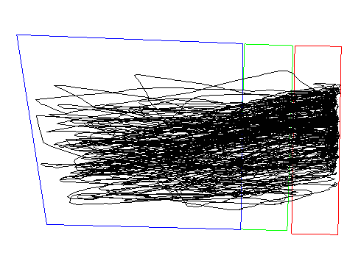 | 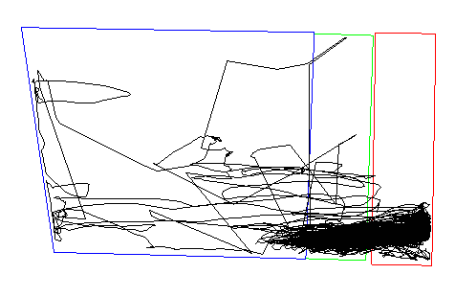 | 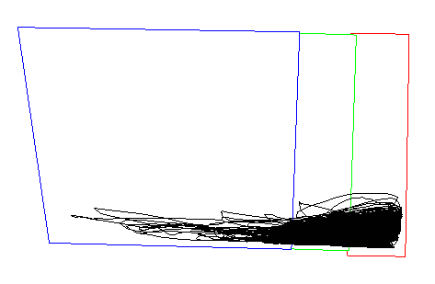 |
| 4 | 5 | 6 |
| 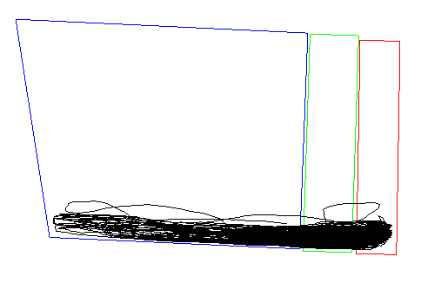 | 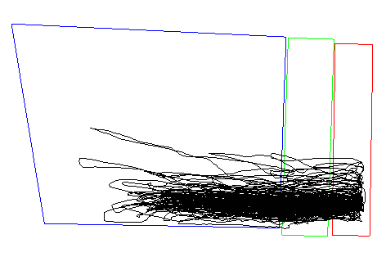 | 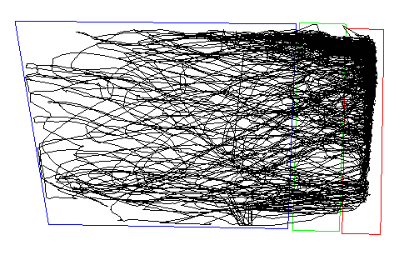 |
| 7 | 8 | 9 |
|  | 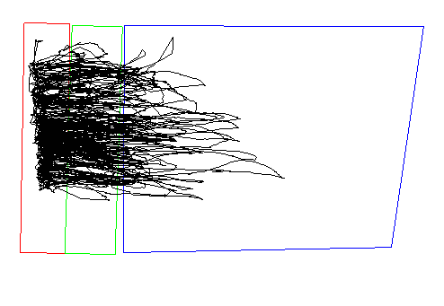 |  |
|  | 10 |  |
| **Mirror Biting Assay : Control + DEX** | | |
| 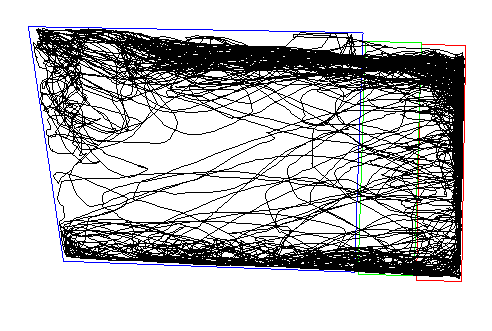 | 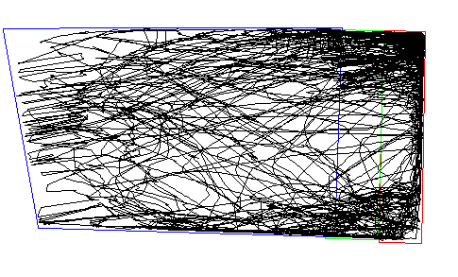 | 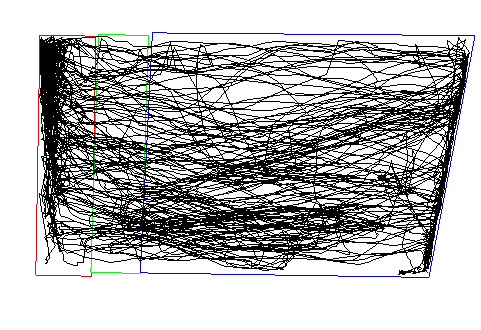 |
| 1 | 2 | 3 |
| 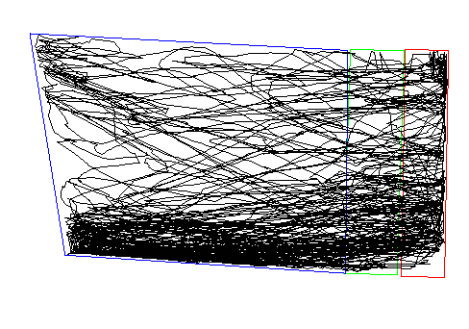 | 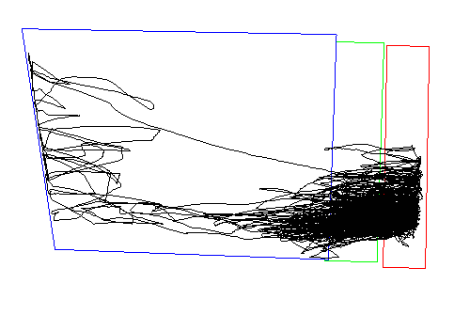 | 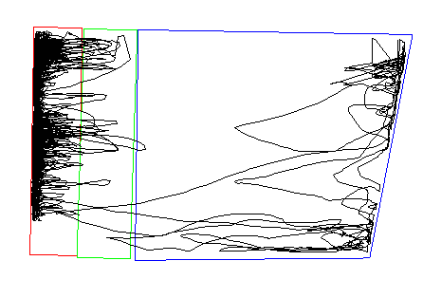 |
| 4 | 5 | 6 |
| 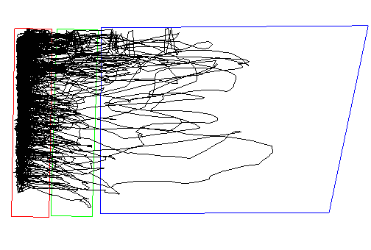 | 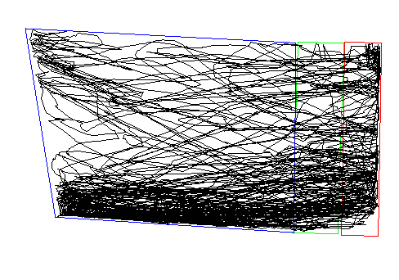 | 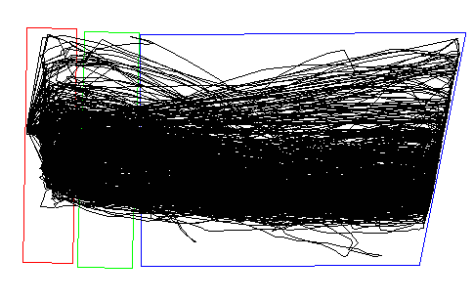 |
| 7 | 8 | 9 |
|  | 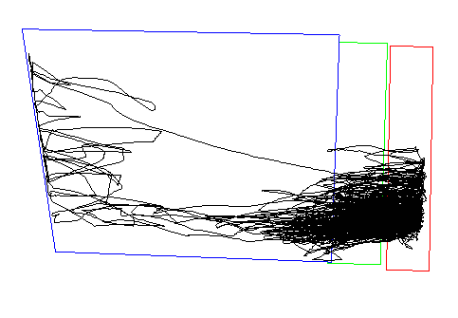 |  |
|  | 10 |  |
